# A tutorial on fitting joint models of M/EEG and behavior to understand cognition

**DOI:** 10.3758/s13428-023-02331-x

**Published:** 2024-02-26

**Authors:** Michael D. Nunez, Kianté Fernandez, Ramesh Srinivasan, Joachim Vandekerckhove

**Affiliations:** 1 Psychological Methods, University of Amsterdam, Amsterdam, The Netherlands; 2 Department of Psychology, University of California, Los Angeles, CA USA; 3 Department of Cognitive Sciences, University of California, Irvine, CA USA; 4 Department of Biomedical Engineering, University of California, Irvine, CA USA; 5 Institute of Mathematical Behavioral Sciences, University of California, Irvine, CA USA; 6 Department of Statistics, University of California, Irvine, CA USA

**Keywords:** Computational modeling, Cognitive modeling, Electroencephalography (EEG), Magnetoencephalography (MEG), Psychology, Neuroscience

## Abstract

We present motivation and practical steps necessary to find parameter estimates of joint models of behavior and neural electrophysiological data. This tutorial is written for researchers wishing to build joint models of human behavior and scalp and intracranial electroencephalographic (EEG) or magnetoencephalographic (MEG) data, and more specifically those researchers who seek to understand human cognition. Although these techniques could easily be applied to animal models, the focus of this tutorial is on human participants. Joint modeling of M/EEG and behavior requires some knowledge of existing computational and cognitive theories, M/EEG artifact correction, M/EEG analysis techniques, cognitive modeling, and programming for statistical modeling implementation. This paper seeks to give an introduction to these techniques as they apply to estimating parameters from neurocognitive models of M/EEG and human behavior, and to evaluate model results and compare models. Due to our research and knowledge on the subject matter, our examples in this paper will focus on testing specific hypotheses in human decision-making theory. However, most of the motivation and discussion of this paper applies across many modeling procedures and applications. We provide Python (and linked R) code examples in the tutorial and appendix. Readers are encouraged to try the exercises at the end of the document.

## Motivation to model

*Joint modeling*, or models that link distributions from multiple modalities, are useful tools for studying cognition (Teller, 1984; Hanes & Schall, 1996; Schall, 2004). There is considerable value to joint modeling in M/EEG measures to behavioral data to answer questions about cognition. Fitting joint models of M/EEG and human behavior have been used to answer diverse questions about cognitive topics such as working memory (Zhang, van Vugt, Borst & Anderson, 2018), reinforcement learning (Frank et al., 2015; Swart et al., 2018), cognitive abilities (Schubert, Nunez, Hagemann, & Vandekerckhove, 2019), and even the study of dyslexia in children (Manning et al., 2021). Schubert et al. (2019) show that neural processing speed, as reflected in stimulus-locked EEG measures, describes variation in cognitive task performance across individuals. In another study, Nunez, Srinivasan, & Vandekerckhove (2015) show that EEG measures of attention can reveal how individual differences in visual attention affect differentiable cognitive components of decision-making.

What do we hope to achieve by finding parameter estimates of joint models of M/EEG and human behavior? We could use these parameter estimates to draw conclusions about a scientific hypothesis, to help differentiate between theories, to find models that best predict human behavior and brain dynamics, or to teach students how to fit models to data. Researchers may also find intrinsic value in using joint modeling of M/EEG versus other neuroimaging modalities such as fMRI due to the high temporal resolution and often a shared scale with behavioral data measured in time, e.g., a shared scale of seconds after a stimulus between M/EEG and response time measures. While the possible goals that could be realized by joint modeling of M/EEG and human behavior are numerous (e.g., see Kording, Blohm, Schrater, & Kay, 2018), most researchers who fit joint models will seek to either (1) test specific hypotheses or differentiate theories in fields such as neuroscience and psychology or (2) maximize prediction of M/EEG signals and/or human behavior. Sometimes these two goals can be simultaneously realized, but maximizing prediction of M/EEG and human behavior is often best achieved with atheoretical approaches based in machine learning (ML) or artificial intelligence (AI). ML and AI are best used in scenarios where maximizing prediction is most important and understanding the cognitive process is not critical, for instance in many brain–computer interfaces (BCIs).

In this tutorial we will focus primarily on joint modeling for testing specific hypotheses. The diversity in *methods* used to perform joint modeling of M/EEG and behavior is large. The studies mentioned above include a variety of methods to perform joint modeling. There are also many related studies that correlate cognitive model parameters to observed M/EEG measures or correlate observed behavioral measures to computational parameters of EEG (e.g., O’Connell, Dockree, & Kelly, 2012; Gluth, Rieskamp, & Büchel, 2013; Jagannathan, Bareham, & Bekinschtein, 2021). Our intention in this tutorial is not to review all cognitive topics (e.g., see Hawkins, Cavanagh, Brown, & Steyvers, 2023) nor all joint modeling techniques of M/EEG and behavior (e.g., see Palestro et al., 2018). Instead, we will focus on clarifying common modeling examples, EEG data collection and analysis, and tools to implement joint models. We will cover experimental design, M/EEG analysis, and behavioral analysis techniques necessary for joint modeling, as well as the modeling itself. Due to our research and knowledge on the subject matter, our examples in this paper will focus on testing specific hypotheses in human decision-making theory. However, all techniques and software presented here can be applied to testing any formal hypotheses involving the relationship of M/EEG to human cognition and behavior.

We expect a diversity in readership of this tutorial from different disciplines. We primarily expect readers trained in two different sub-fields, namely, cognitive neuroscience and mathematical psychology. Therefore, some readers may find certain sections introductory. Readers trained in cognitive neuroscience should place increased focus on “Models to describe joint data” and “Implementing model-fitting procedures and estimating parameters”, which give the motivation and techniques for modeling, while readers trained in mathematical psychology should focus on “Experimental manipulations and experimental design” and “Collection and preprocessing of M/EEG for joint modeling” that provide experimental motivations and the theory and practice of M/EEG. Other readers may need to read the entire tutorial carefully, as well as follow the references in *Further readings* (located at the end of the paper) corresponding to each main section if looking for more background or depth on a certain topic. Finally, we encourage readers to read and run the provided example scripts in either Python (https://github.com/mdnunez/pyhddmjags) or R (https://github.com/kiante-fernandez/Rhddmjags) and work through the *Exercises* at the end of the document.

### One example topic: Decision-making

**Fig. 1:**
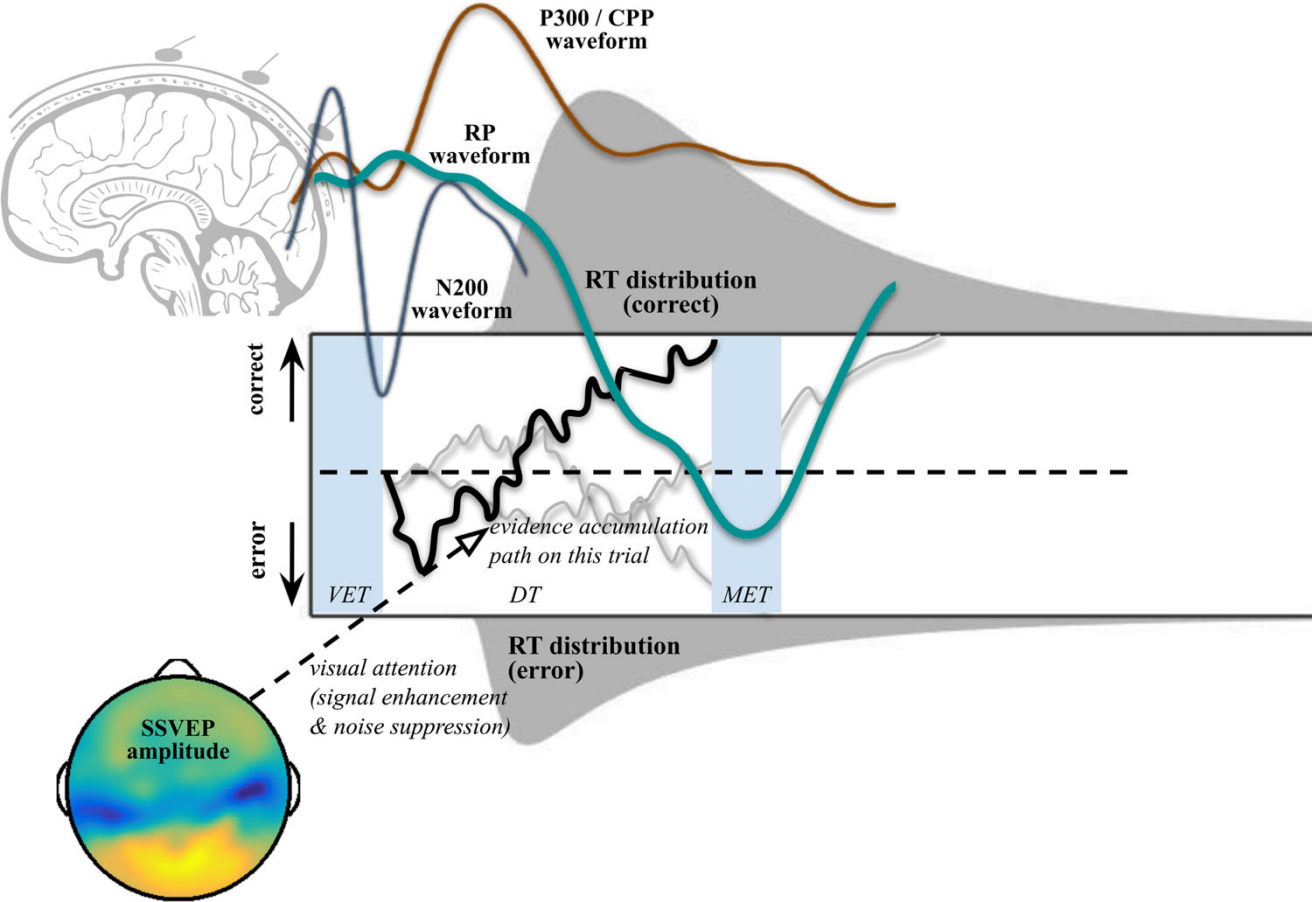
A theoretical representation of some modeling studies to discover cognitive mechanisms of decision-making using *neurocognitive* modeling of EEG and human behavior during decision-making tasks. *Bold text* represents observed data (EEG measures or human behavioral data) while italic text represents derived cognitive parameters that can be estimated through joint modeling. Event-related potentials (ERPs; represented by three waveforms beginning at the cartoon image of the brain in the *top right*) and frequency-domain EEG measures (*bottom left*: EEG amplitudes that were spline-interpolated between electrodes on a flat representation of the human scalp) have been used in joint modeling to understand human cognition in the context of neurocognitive drift-diffusion models (NCDDMs). Human behavioral data such as choice-RTs (response time distributions shown for correct responses, *top*, and error responses, *bottom* flipped) are also used to fit NCDDMs and drift-diffusion models (DDMs). In NCDDMs, like DDMs, correct and error responses are described after enough cognitive evidence is reached, representing by the cognitive evidence accumulation passing one of two boundaries during decision time (this trial represented as a *black line* with *two other grey lines* representing other simulations from the same process that describe response times and possibly EEG potentials). Particular ERPs of interest are N200, P300 / CPP, and RP waveforms. N200 waveforms are thought to reflect visual encoding time (VET) and the onset of evidence accumulation (Nunez et al., 2019a). The P300 or centro-parietal positivity (CPP) are thought to reflect decision time (DT) and possibly the evidence accumulation process itself (O’Connell et al., 2012; Kelly & O’Connell, 2013; O’Connell et al., 2018; van Ravenzwaaij et al., 2017). The readiness potential (RP) is a motor related preparatory signal thought to reflect DT and motor execution time (MET) under certain experimental conditions (Lui et al., 2021). Steady-state visual evoked potentials (SSVEPs) can be used to estimate visual attention and in particular, signal enhancement and noise suppression that could affect the rate and variance of evidence accumulation (Nunez et al., 2015). A table of related questions and other neurocognitive work using DDMs can be found in Table 1

One topic of interest is whether specific M/EEG signals reflect cognitive components of decision-making. This has been the focus of a large body of previous work (e.g., see O’Connell, Shadlen, Wong-Lin, & Kelly, 2018) including our own work (see Fig. 1, Lui et al., 2021). This work has led to specific testable questions that can be answered with joint modeling of M/EEG data, response time data, and choice data from tasks in which participants make simple decisions. However, before we discuss this example of joint modeling work, let us first briefly review a key theory of decision-making.

Sequential sampling models assume that humans and animals accumulate evidence for a particular choice over the course of a decision by *sampling* from external or internal evidence. This *evidence* for a decision is usually considered a cognitive representation or a direct neural representation (e.g., changing firing rates of neurons over time). Simulating and fitting sequential sampling models are particularly useful for understanding quick decisions on the scale of seconds. These models make predictions about the time course of decision-making, while other decision-making models, such as signal detection theory (SDT; Hautus, Macmillan, & Creelman, 2021) do not make any predictions about the time course of decision-making. Drift-diffusion models (DDMs; Ratcliff, 2018) are a particular class of *sequential sampling* models that assume a Wiener process of evidence accumulation. A Wiener process of evidence accumulation is a random walk process with an infinitesimal (infinitely small) time step (see middle of Fig. 1). However, note though that DDMs and associated model variants are often used due to the models’ mathematical utility, rather than a theoretical belief that a time step of evidence accumulation should be infinitesimal in the brain.

Sequential sampling theory leads naturally to specific testable questions that can be answered with joint modeling of M/EEG data. Do time-averaged event-related potentials (ERPs) encode the demarcation point between visual encoding and evidence accumulation (Nunez, Gosai, Vandekerckhove, & Srinivasan, 2019a)? Do motor preparation signals over the motor cortex track evidence accumulation time (Lui et al., 2021)? How do EEG measures of visual attention affect the decision-making process, and in what precise way does visual attention affect different computational components of decision-making (Nunez et al., 2015; Nunez, Vandekerckhove, & Srinivasan, 2017)? A table of related questions that can be answered with neurocognitive models using DDMs can be found in Table 1. Multiple analysis methods exist to help answer these questions with EEG and behavior (see Bridwell et al., 2018). However, we have preferred to use the implementation of joint modeling of EEG and human behavior to understand data from participants who performed hypotheses-differentiating experiments.

**Table 1:** A table of example hypotheses that could be tested directly using neuro-cognitive drift-diffusion models (DDMs) of M/EEG and behavior

Cognitive mechanism	Computational role	Neural signature	DDM parameters	References
Visual evidence accumulation	Evidence accumulation rate	P300 slopes	$\delta$	(Philiastides, Ratcliff, & Sajda, 2006; Ratcliff, Philiastides, & Sajda, 2009; Philiastides et al., 2014; Twomey et al., 2015; van Ravenzwaaij et al., 2017; Kohl, Spieser, Forster, Bestmann, & Yarrow, 2020)
Subjective-value evidence accumulation	Evidence accumulation rate	Gamma (46-64 Hz) power	$\delta$	(Polanía, Krajbich, Grueschow, & Ruff, 2014)
Figure-ground segregation	Visual encoding time (VET)	N200 latencies	$\tau$ / $\tau ^{v}$	(Loughnane et al., 2016; Nunez et al., 2017; Nunez et al., 2019a; Ghaderi-Kangavari, Rad, Parand, & Nunez, 2022; Ghaderi-Kangavari, Parand, Ebrahimpour, Nunez, & Amani Rad, 2023a)
Motor execution	Motor execution time	Beta (15–25 Hz) desynchronization	$\tau$ / $\tau ^{m}$	(Crone et al., 1998; McFarland, Miner, Vaughan, & Wolpaw, 2000)
Motor cortex preparation	Motor evidence accumulation	Readiness potentials	$\delta$	(Gluth et al., 2013; Lui et al., 2021)
Speed–accuracy tradeoff	Changing neural threshold	Theta (4–7 Hz) power	$\alpha$	(Cavanagh et al., 2011; Frank et al., 2015)
Strategy adjustment	Changing neural threshold	Contingent negative variation	$\alpha$	(Boehm, van Maanen, Forstmann, & van Rijn, 2014)
Prestimulus activation	Bias	Occipital EEG amplitude to predict choice	$\beta$	(Bode et al., 2012)
Visual attention	Variability in evidence (diffusion)	Steady-state visual evoked potentials	$\delta$, $\varsigma$	(Nunez et al., 2015; Rangelov & Mattingley, 2020)
Attentional gating	Internal neural noise	Alpha (8–12 Hz) power	$\varsigma$	(Pfurtscheller, Stancák, & Neuper, 1996; Jensen & Mazaheri, 2010; Klatt et al., 2020)

Each hypothesis is derived directly from existing literature in the fields of cognitive neuroscience and model-based cognitive neuroscience. Each *cognitive mechanism* is associated with a specific *computational role* in the human brain that is measured with a M/EEG *neural signature* that is hypothesized to be reflected in the relationship with a cognitive parameter of a DDM. $\delta$ refers to the drift rate parameter, $\tau$ refers to the non-decision time, $\tau ^{v}$ refers to visual encoding time (a component of non-decision time) $\tau ^{m}$ refers to motor execution time (another component of non-decision time) $\alpha$ refers to the boundary separation parameter, $\beta$ refers to the initial bias parameter, $\varsigma$ refers to the diffusion coefficient (undiscussed in this text but discussed in Ratcliff et al., 2016; Nunez et al., 2017, and elsewhere)

### One example question: Do EEG signals encode sequential-sampling of evidence?

One more specific question is whether EEG signals encode a sequential-sampling of evidence, such has been found in single neurons and neural populations within intracranial recordings of the lateral intraparietal (LIP) cortex and superior colliculus (SC) of the macaque brain during single experimental trials (Roitman & Shadlen, 2002; Shadlen & Kiani, 2013; O’Connell et al., 2018; Jun et al., 2021). EEG signals time-locked to specific events such as the onset of a visual stimulus, e.g., the P300 / centro-parietal positivity (CPP) waveform, and EEG signals time-locked to the response, e.g., the readiness potential (RP), have been proposed to be related to evidence accumulation and the timing of decisions (O’Connell et al., 2012; Gluth et al., 2013; Twomey, Murphy, Kelly, & O’Connell, 2015; van Ravenzwaaij, Provost, & Brown, 2017; Lui et al., 2021). We expect future joint modeling work will further help differentiate whether these signals are exactly encoding evidence accumulation, correlated processes, or mixtures of signals (Philiastides, Heekeren, & Sajda, 2014). Related questions that can be answered with joint modeling work are: (1) what EEG preprocessing steps and recording procedures should be used to best extract evidence accumulated related signals? and (2) for what specific conditions and task paradigms these signals encode evidence accumulation? In this paper, we show how combining these specific EEG signals and behavioral data in neurocognitive modeling will lead to better and more extensive knowledge of individual differences and single-trial estimates of human cognition. In our example, methods presented in this paper, we build models and present model-fitting procedures that can best answer the question of whether EEG signals encode a sequential-sampling of evidence.

## Models to describe joint data

### The basic terms of modeling

*Cognitive models* include parameters of psychological processes that describe human (or animal) behavior. These models are often used to describe behavior in psychological experiments or natural environments, and these models are often developed by researchers in the scientific field of mathematical psychology. A *parameter* of a cognitive model is a variable that can take a pre-specified range of values that describe data, and multiple parameters of a model are usually required to describe data. The parameters of cognitive models often directly relate to unobserved psychological concepts such as memory capacity or general cognitive ability (Lee et al., 2019; Schubert et al., 2019). Signal detection theory (SDT) that explains choice and accuracy data could be considered a cognitive model since it contains two parameters that describe both the ability and choice bias of a human participant (Hautus et al., 2021). Another example are drift-diffusion models (DDMs) of choices and response time (RT) data during human decision-making, which contain cognitive parameters that describe speed–accuracy trade-offs, speed of evidence accumulation for one choice or another, and decision biases (Ratcliff, Smith, Brown, & McKoon, 2016). The cognitive interpretations of parameters of new cognitive models should be tested by experimentation with differentiating experimental conditions (discussed below). However, some widely used cognitive models, such as SDT and DDMs, have parameters whose cognitive interpretations are now widely accepted by researchers due to the results of multiple experimental studies. For instance, Voss, Rothermund, and Voss (2004); Dutilh et al. (2019) generally found that parameters of DDMs (namely speed–accuracy tradeoff parameters, non-decision time parameters, evidence accumulation rate parameters, and evidence bias parameters) that describe human choice and response time are all manipulated in expected directions by proper experimental conditions. Although these experimental manipulations are not perfect, and they often affect other cognitive parameters (Dutilh et al., 2019), these prior results allow us to draw conclusions in new data while assuming some of these parameters map onto the expected cognitive function.

Cognitive models are sometimes defined differently from *computational models*. Computational models often focus on modeling brain dynamics with parameters that have specific neural correlates (Blohm, Kording, & Schrater, 2020; Glomb et al., 2021) (a short discussion of M/EEG generators can be found in the Discussion section). Typically computational models are used in the scientific field of computational neuroscience. Though computational models do not necessarily include parameters with interpretable psychological processes. However, because computational models may describe human behavioral data, the term “computational model” is sometimes used more broadly to encompass cognitive models as well (e.g., Wilson & Collins, 2019).

*Neurocognitive models* are joint models of brain activity and human behavior that seeks to combine cognitive modeling with necessary links between (1) brain dynamics as measured by or derived from M/EEG, (2) cognition and other psychological concepts expressed as formal models, and (3) human behavioral data. As a generalization, with neurocognitive modeling we seek to understand how macro-level neurophysiology (as measured by scalp-recorded EEG, MEG, or even depth-recorded EEG) encodes human cognition which gives rise to human behavior. For instance, evidence accumulation is a cognitive process during decision-making, but may also have direct neural correlates in EEG (Forstmann, Ratcliff, & Wagenmakers, 2016; O’Connell et al., 2018; Lui et al., 2021), and thus drift rates of DDMs could describe choice-RT distribution shapes, evoked EEG potentials, or both simultaneously. Thus, these *neurocognitive* models can be used to develop and test theories in both psychology and neuroscience. Specifically we focus in this paper on how modeling can be used to directly test hypotheses which involve observed M/EEG dynamics, human cognition, and human behavior. Note that *neurocognitive modeling* is less defined than *joint modeling*, as is defined more specifically for certain classes of neurocognitive models (see Turner, Forstmann, Love, Palmeri, & Van Maanen, 2017; Palestro et al., 2018). In the next section, we describe three examples of neurocognitive models, and Models 2 and 3 could be considered joint models in the labeling scheme of Turner et al. (2017) and Palestro et al. (2018).

When *simulating* models, parameters are fixed to certain values and a model generates synthetic data using programs such as R and Python that could be compared to real data. For instance, a neurocognitive model with multiple user-defined parameters could generate synthetic (but informative) EEG potentials, choices, and response times. To *fit* a model is to discover a set of parameter estimates that best describe known data given the model architecture and assumptions. *Fitting* a model using Bayesian methods means finding parameter uncertainties from which parameter estimates can be derived. Fitting models to data is a useful method to test hypotheses either (1) by directly estimating and then evaluating parameters (e.g., compare parameter estimates across experimental conditions) or (2) comparing multiple models’ ability to describe data.

### Translating neurocognitive theory into mathematical models

Often modeling involves simplifying the broader mathematically defined theories of the brain and human behavior to fit data accurately and efficiently. In this way theory, or an approximation of theory, can be used to answer questions directly. Fitting models to EEG and behavioral data requires (1) knowledge of cognitive or neurocognitive theory, and (2) effort to quantitatively formalize the hypotheses to be tested in the context of theory. Good theory should be quantitatively defined (Oberauer & Lewandowsky, 2019a), and the best theory is precisely mathematically defined (Guest & Martin, 2021). Although many other qualifications may be needed for good theory, these are discussed elsewhere (e.g., van Rooij & Baggio, 2020).

A model should be able to be written as a series of mathematical equations and statistical distributional statements that describe the data. Let us assume that a participant, Roos, wore an EEG cap while playing a simple video game where she made a correct or incorrect answer on multiple trials of the game. Researchers extracted EEG and behavioral data from the data collection hardware and are now interested in how the centro-parietal positivity (CPP) rise over time within a trial (e.g., the CPP “*slope*”) influences accuracy and response times (RTs). Note that the CPP slope is a signal in the EEG that is found after the onset of visual stimuli, and is thought to be a reflection of the computation of visual evidence in the brain (O’Connell et al., 2012, 2018). Specifically the researchers are interested in how three measures from Roos are related: CPP slopes, accuracy, and RTs. The researchers obtain observations of each of the three measures on every trial of the game. One simple joint model could just assume that RTs and accuracy are influenced by the CPP slope. Thus, a simple model (Model 1) would just be two attached models, linear regression and logistic regression. Let CPP slope be denoted by the variable *c*, accuracy be denoted by *x*, and response times be denoted by *r*. Note that accuracy *x* can be either 0 or 1, and that *x*, *r*, and *c* can vary on every trial *i* and by participant *j*. The parameters of the model can change by participant *j*. Researchers can then fit this model to Roos’ data as well as other participants.

1 $$\begin{aligned}&r_{ij} \sim Normal(\theta _{0j} + \theta _{1j} c_{ij} , \ \sigma ^{2}_{j}) \end{aligned}$$

2 $$\begin{aligned}&x_{ij} \sim Bernoulli(p_{j}) \end{aligned}$$

3 $$\begin{aligned}&\log \Big ( \frac{p_{j}}{1-p_{j}} \Big ) = \gamma _{0j} + \gamma _{1j} c_{ij} \end{aligned}$$

Note that the symbol $\sim$ denotes *distributed by*, so that response times *r* come from a normal distribution with a mean parameter that changes with a linear function of CPP slopes *c* and variance $\sigma ^2$. This equation represents simple linear regression. The second distributional statement and the last equation represent logistic regression. We picked the logit (“log-odds”) function $\textit{logit}(p) = \log (\nicefrac {p_{j}}{1-p_{j}})$ within the logistic regression framework, although we could use any function that maps probabilities bounded from 0 to 1 to the continuous $(-\infty , \infty )$ scale. The $\theta$ and $\gamma$ parameters in this model provide the relationship of EEG measure to behavior. Note that we can test whether the $\theta _{1}$ and $\gamma _{1}$ parameters are near zero in order to test many hypothesized relationships between CPP slope and behavior. Alternatively, we could compare the fit of the behavioral data in this model to a model where the EEG does not influence the data generators. In a comparison model, response times and accuracies would be described only by the mean and probability parameters (e.g., setting $\theta _{1}$ and $\gamma _{1}$ both to 0 before model fitting). Note that Model 1 does not assume EEG reflects any *particular* type of cognition or computation. However, our example question is more specific about the type of computation and cognition the CPP slope could reflect. Namely, that CPP slope reflects evidence accumulation speed. Model 1 assumes that any relationship is linear between CPP slopes and response times as well as CPP slopes and log odds of accuracies. Fitting Model 1 will likely yield information about the existence of a CPP–behavior relationship because linear and logistic regression are useful in finding relationships where there is *some* true relationship between variables. Model 1 would be particularly helpful if we wanted to test if there was any possible relationship in an exploratory analysis.

**Fig. 2:**
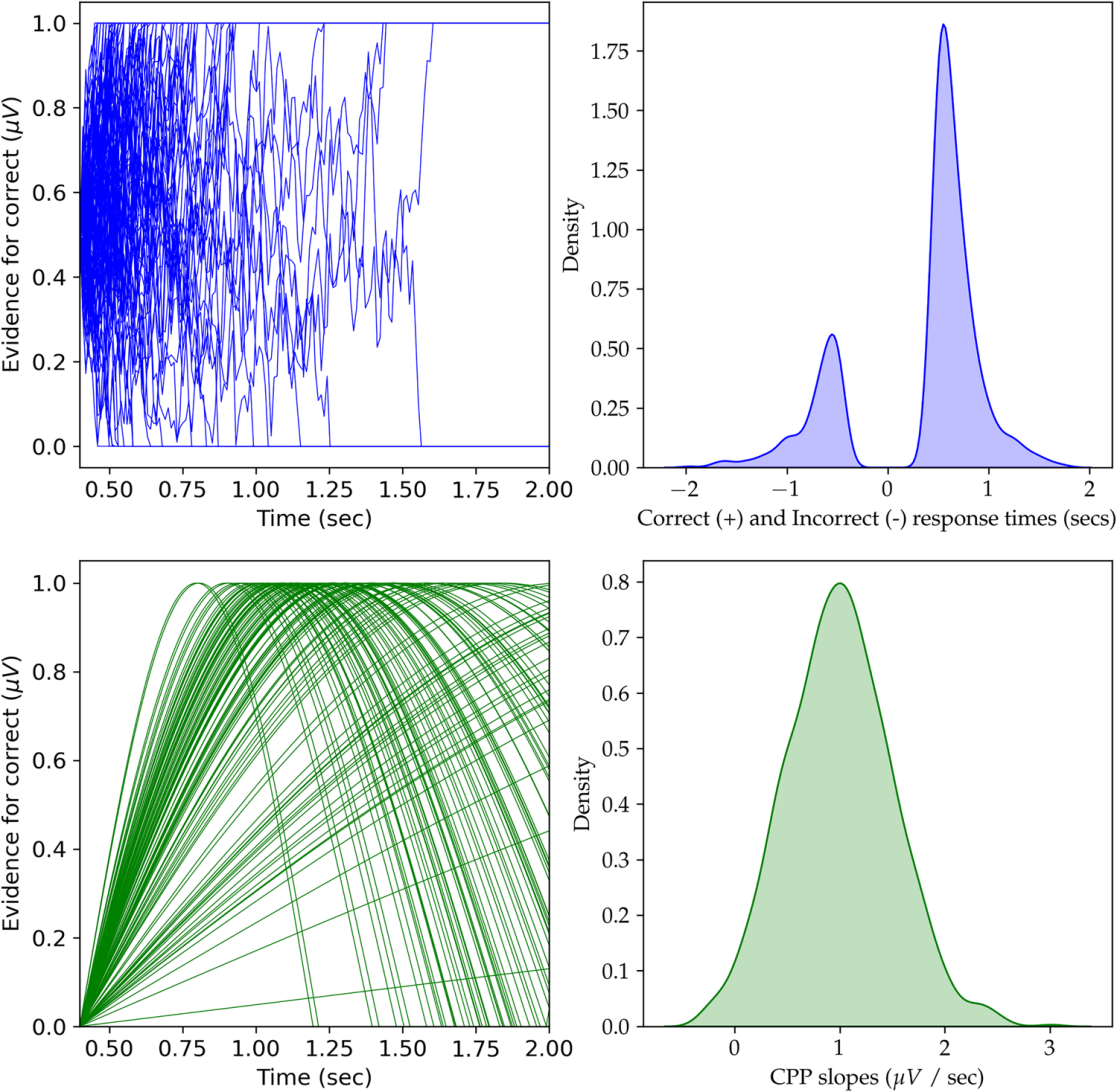
Diagnostic plots of the simulation of Model 3. We simulated Model 3 using the provided Python code with 1000 trials for one participant. The *top left figure* is the simulated evidence accumulation paths from a Wiener process that reaches one of two boundaries to make a decision. The *top right figure* is the estimated density of incorrect and correct response times, with incorrect response times plotted as negative response times. The *bottom left figure* is the simulated CPPs on single-trials using a simple sine wave. The *bottom right figure* is approximated density of the simulated CPP slopes. This density should be approximately normal because of our modeling assumption for CPP slopes

Researchers instead might want to directly test whether the CPP slopes reflect specific cognitive components of decision-making that describe both the accuracy and response time jointly. A second model (Model 2) could then test simple linear relationships to cognitive parameters. In particular, we might be interested in how CPP slopes describe the drift-rate parameters $\delta$ and the non-decision time parameters $\tau$. The drift rate $\delta$ reflects the mean rate of evidence accumulation for each infinitesimal time step of a Wiener process (Ratcliff et al., 2016). Non-decision time $\tau$ on every trial is any time in a response time not due to a Wiener process (such as visual encoding time; VET). Let’s assume that $\delta$ and $\tau$ can vary on every experimental trial. We also assume that other parameters of a DDM do not vary with CPP slope and are fixed across trials, namely: the boundary separation parameter $\alpha _{j}$ which describes the amount of relative evidence to make a correct choice, the initial bias parameter $\beta _{j}$ towards the correct choice before evidence accumulation occurs, and trial-to-trial variability in drift rate, $\eta _{j}$, that is not due to CPP slope variability. This DDM describes both response times *r* and choices *x* per participant *j* and trial *i*.

4 $$\begin{aligned}&(r_{ij},x_{ij}) \sim DDM(\delta _{ij} ,\tau _{ij}, \alpha _{j}, \beta _{j}, \eta _{j}) \end{aligned}$$

5 $$\begin{aligned}&\delta _{ij} = \xi _{0j} + \xi _{1j} c_{ij} \end{aligned}$$

6 $$\begin{aligned}&\tau _{ij} = \lambda _{0j} + \lambda _{1j} c_{ij} \end{aligned}$$

Note that the parameters $\xi$ and $\lambda$ have different meanings in this model with embedded cognitive components compared to the $\theta$ and $\gamma$ parameters in the previous model. The parameters $\xi$ and $\lambda$ are the intercept and effect parameters of the CPP slopes on the evidence accumulation rate and non-decision time, respectively. We will call this model a *neurocognitive* model because it assumes particular types of cognition during decision-making and contains neural data, in addition to behavioral data. We have fit this class of model, which assumes single-trial EEG measures describe single-trial DDM parameters, in previous work (Nunez et al., 2017, 2019a).

Another class of neurocognitive models involves describing the cognitive parameters through the EEG measures themselves. For instance, we can directly test the underlying computational role of the CPP slope in cognition by testing how well the following model fits the data once parameters are estimated and how well this model predicts new data. In **Model 3**, the mean of each trial’s CPP slope *c* is described by the drift rate for each participant *j*.

7 $$\begin{aligned}&(r_{ij},x_{ij}) \sim DDM(\delta _{j} ,\tau _{j}, \alpha _{j}, \beta _{j}, \eta _{j}) \end{aligned}$$

8 $$\begin{aligned}&c_{ij}\sim Normal(\delta _{j} , \ \sigma _{j}^{2}) \end{aligned}$$

such that five cognitive parameters of a DDM, $\delta$, $\tau$, $\alpha$, $\beta$, and $\eta$ vary by participant *j*. Note that one of those cognitive parameters, the mean rate of evidence accumulation across trials $\delta$ is also a *computational parameter*, a parameter that describe neural data in a *computational model* define above, that describes the CPP slope on every trial *i* and each participant *j*. There is one more additional computational parameter in this model that describes the observation noise of the CPP slope on every trial *i*, parameter $\sigma$ for each participant *j*. This model is similar to, although more specific than, a model previously used by van Ravenzwaaij et al. (2017).

Better neurocognitive models might extend Model 3 by describing the CPP slopes from a function of multiple cognitive parameters, for instance. Other models could contain computational parameters that reflect brain dynamics that are described by cognitive parameters. Different modeling strategies are discussed by Turner et al. (2017). All of the example models and other joint models can be simulated through the equations listed and by choosing a set of values for parameters. We can build a variety of neurocognitive models to test specific theories.

### The importance of model simulation

Model simulation is usually the first step in generating new models, and can be helpful in understanding the neurocognitive theory. Model fitting intrinsically makes many assumptions about the data. Joint modeling of M/EEG and behavior will make assumptions about cognitive and computational processes in the brain and implicit assumptions about what cognitive and computational processes are *not* occurring. These assumptions will affect parameter fitting results significantly. It is therefore imperative that the researcher understand what assumptions they are making and how those assumptions can be violated.

The best way to explore these assumptions is through the two-step process of (1) simulation of models using different model types and realistic parameter ranges and then (2) fitting all the simulated data using the specific model and fitting procedure to be used in the analysis of real data. Simulations can show the researcher under which conditions the fitting procedure can break or produce spurious results. Simulation of models, as opposed to fitting of models, is also one way to formally define the underlying *theory* to be tested, in that it is quantifiable, and perhaps complex, but rigorously defined (Guest & Martin, 2021).

To simulate joint models of M/EEG, it is best to write the model using existing statistical libraries with coding languages such as Python or R. We reproduced one possible simulation of Model 3 here. A code snippet of Model 3 is provided in Code Block 1 with associated full Python and R code located at https://github.com/mdnunez/pyhddmjags/blob/master/simpleCPP_sim.py and https://github.com/kiante-fernandez/Rhddmjags/blob/main/R/Rhddmjagsutils.R, respectively, as of July 2023. We first generate random values of all parameters for one participant. We then simulate data for all trials *i* for this participant from those parameters within the two equations of Model 3 in a for loop. Our code simulates the model directly from an approximation of the Wiener process in line 9 (specifically approximated using the Euler method, see Brown, Ratcliff, & Smith, 2006). The approximation can be thought of as a random walk process with a very small time step that loops over nsteps (line 8), until the random walk passes the upper boundary alpha (line 11) or lower boundary 0 (line 16). This is especially useful for observing simulations of the evidence paths on every trial (see top left of Fig. 2). Our code also simulates the CPP observations themselves from a sine wave (line 6), commonly used for simulating oscillatory signals (Cohen, 2014), with 1/4 of the period of the sine wave being the CPP slope (see bottom left of Fig. 2). Protocols for simulating EEG data range in biological and physical plausibility, however, sine waves are a common starting point for simulating EEG activity (Cohen, 2014; Hagen et al., 2022; Næss et al., 2021), see also the section on modeling of M/EEG generators in the Discussion of this paper.
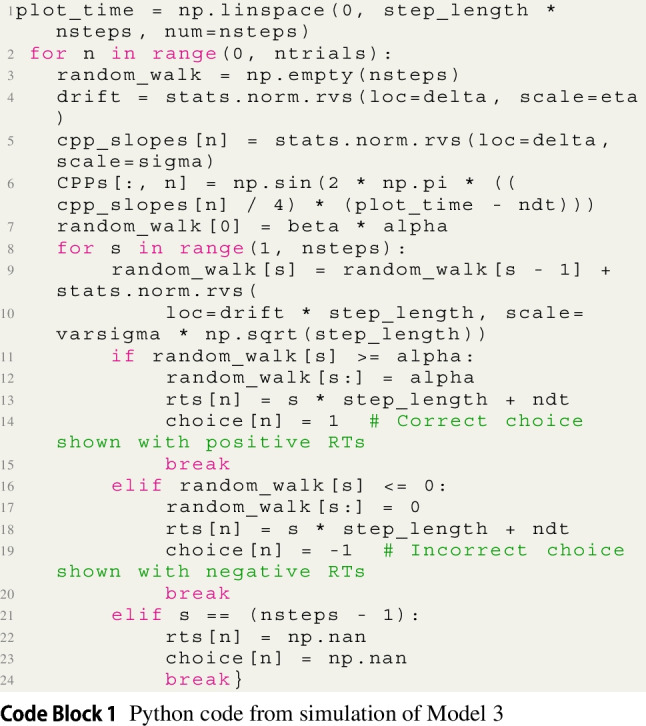


When simulating a model it is useful to plot elements of the model itself. For instance in Fig. 2 we have plotted dynamics of the model itself, namely the CPP waveforms on single-trials and the evidence paths themselves. We have also plotted the distributions of both the choice response times and the CPP slopes. We can also choose to plot other diagnostics such as the cumulative distribution function, and return specific statistics about our simulated data (e.g., mean, median, maximum, and minimums). In this way we can make sure our model, and quantitatively defined theories, are logical and could describe real data. Note however that we may not observe everything about our model in real data nor be able to estimate everything about our model from the data. For instance, we may never observe the cognitive evidence paths themselves (top left of Fig. 2) in real data. Later, after we discuss model fitting, we will discuss how we can also use simulations to test parameter recovery. We will show that *fitting* Model 3 to data is indeed useful.

## Experimental manipulations and experimental design

The goal of experimentation should be to design experimental conditions that best answer scientific questions. In this section, we will focus on experiments that are optimally designed to produce data for use with joint modeling. One particular *neurocognitive* theory suggests that a particular trial-averaged EEG signal, the CPP, in response to the onset of a picture on a computer screen is expected to be a neural signature of evidence accumulation during decision-making based on that picture. For example, the CPP reflects evidence accumulation to decide whether a noisy picture is a face or a car (e.g., Ostwald, Porcaro, Mayhew, & Bagshaw, 2012). This is a theory suggested by experimental and theoretical work by O’Connell et al. (2012, 2018), previously mentioned. Thus, we should design experiments to collect M/EEG and behavioral data and/or pick an existing data set that (1) best tests the hypothesis and (2) test the limits of this hypothesis.

### Hypothetical experiment 1

We know that drift rates in perceptual decision-making tasks are affected by perceptual difficulty (Voss et al., 2004; Dutilh et al., 2019). Therefore, if CPPs are signatures of the computational mechanism of evidence accumulation in the brain, we expect drift rates and CPP slopes to both change across perceptual difficulty conditions, and specifically maintain the same relationship across many experimental conditions of various difficulty. For instance participants could perform a random dot motion (RDM) task with many different levels of perceptual difficulty across trials, a replication of work by Kelly and O’Connell (2013). An RDM task is a task in which a field of moving dots appears on each experimental trial, with a (typically small) percentage of dots moving in a specific direction (e.g., see Newsome & Pare, 1988; Gherman & Philiastides, 2018). Typically participants must choose between one of two directions. If all the dots moved together on the screen, the task would be too easy and would not result in different performance across conditions, not optimally testing our neurocognitive theory. Therefore, the other percentage of dots typically move completely at random with no orientation information. The percentage *coherence* of dots describes the percentage of dots that move in the correct orientation. Therefore, let us imagine a task in which trials are intermixed with 32%, 16%, 8%, 4%, and 0% coherence values. Note that the difficulty of the coherence values will depend, among other factors, on the size of the dots in the stimulus and the size of the entire stimulus for the participant.

We can fit the data from Experiment 1 to Model 2 for each experimental condition *k*, allowing each parameter and data type to vary by condition *k*. By comparing parameter estimates of the slope parameter $\xi _{1jk}$ across the two conditions, we can test whether there is a fixed linear relationship between the slope of the CPP and the drift rate in all experimental conditions, even when other parameters such as the drift-rate $\delta$ itself change across experimental conditions. Specifically, we could develop statistical tests to test whether $\xi _{1j1} = \xi _{1j2} = \xi _{1j3} = \ldots = \xi _{1jK}$ from the resulting parameter estimates in all participants, for instance, we could analyze posterior distributions of these parameters to calculate posterior probability or Bayes factors (see below). Alternatively, we could compare how two models explain the data and predict new data. For instance, we could compare Model 2A to Model 2B, where Model 2A has fixed effect parameters $\xi _{1j}$ across conditions and Model 2B has effect parameters $\xi _{1jk}$ that are free-to-vary across conditions.

The number of trials and participants to collect in order to answer this question should depend on a *power analysis* (not to be confused with the concept of *EEG power* in the next section). We define a *power analysis* as any simulation-based or statistical theory-based analysis that will give an estimate of either the Bayesian or classical probability of finding true effects (or true null effects) for different sample sizes for specific analyses and joint models. For this particular experiment, we recommend fitting the models in simulation (see section *Parameter recovery of simulated models*) under the simulated truth where $\xi _{1j1} = \xi _{1j2} = \xi _{1j3} = \xi _{1j4} = \xi _{1j5}$ for five simulated conditions with intercept parameters $\xi _{0jk}$ and $\tau _{0jk}$ of Model 2 that change over the experimental conditions *k*. The ultimate planned analysis can then be run on the simulated data with different numbers of trials and participants. For our particular experimental question, we expect the number of trials to be more important than the number of participants (however participant-specific parameters could also be varied in simulation). Power analyses are always recommended over rules-of-thumb because each experimental question and modeling plan will necessitate different trial numbers. We expect that at least 100–500 trials per condition are necessary (Lerche, Voss, & Nagler, 2017). We skipped such analyses for this tutorial for the sake of brevity, and encourage readers to work with the provided simulation code.

### Hypothetical experiment 2

The first hypothetical experiment demonstrates a simple perceptual manipulation intended to drive cognition that best answers our hypothesis. In the second hypothetical experiment, we propose an intervention indented to drive the brain response using transcranial direct current stimulation (tDCS). While Experiment 1 is a well-established experimental manipulation, the intervention in Experiment 2 may or may not have any effect on the participants’ brain response, cognition, and behavior (Chrysikou, Berryhill, Bikson, & Coslett, 2017; Mendes et al., 2022). However, Experiment 2 is a useful example to show that proper control conditions are often necessary to test a hypothesis using joint modeling.

Because of the aforementioned theory, we might expect tDCS during a visual decision-making task to affect both CPP slopes *c* and evidence accumulation rate parameters (i.e., *drift rates*) $\delta$ in DDMs estimated from human behavior. A strong hypothesis is that we expect tDCS to affect both CPP slopes and drift rates equally since the theory is that the CPP is a signature of the computational mechanism of evidence accumulation in the brain. Thus, tDCS could test the limits of the theory of the CPP reflecting evidence accumulation. We should at least have both (1) an experimental condition and (2) a proper control condition in which stimulating tDCS electrodes are applied to the participant’s head.

In tDCS work an experimental control is often a sham condition in which tDCS is turned on then off after a ramping period before a block of experimental trials. This sham condition seeks to achieve the sensation of tDCS stimulation by the participant for a block of experimental trials, but to not actually stimulate during those trials (e.g., see Au et al., 2021). We propose an experimental design with one experimental condition (1) in which the experimenters stimulate brain areas expected to be involved in decision-making (perhaps placing stimulation electrodes on the scalp over parietal cortices), and a control condition (2) in which experimenters stimulate brain areas not expected to be involved in decision-making, say placing tDCS electrodes on the scalp over the temporal cortex. We could also include a different sham condition, another experimental control, (3) in which tDCS electrodes do not stimulate the brain but current is still injected into the body, for instance placing and activating tDCS electrodes over the neck musculature in the back of the head.

We could again fit Model 2B to this data and analyze the results in a similar way to the previous experiment. For instance, suppose we fit the data to Model 2B and subsequently observe that both CPP slopes *c* and drift rates $\delta$ are significantly increased in condition (1) compared to sham condition (3), and that the two effect parameters of the model are equal, $\xi _{1j1} = \xi _{1j3}$. We also observe that only drift rates $\delta$ are significantly increased in condition (2) compared to the sham condition (3) and CPP slopes *c* are similar in both conditions, resulting in $\xi _{1j1} = \xi _{1j3} > \xi _{1j2}$. A simulation of this scenario and a simplified fitting procedure for Model 2B is included in https://github.com/mdnunez/pyhddmjags/blob/master/model2b_experiment2.py. These results would be evidence that the CPP slope reflects evidence accumulation only in specific conditions. For instance, this might suggest the CPP only reflects visual evidence accumulation in the parietal cortex, and is not the brain-wide cognitive phenomena thought to be reflected in decision-making behavior. Of course, we would need further experimentation to test this new, more specific theory. We should also build a new joint model to better reflect our new neurocognitive theory.

### Theoretically informed experiments

While we should be able to *simulate* joint models that account for many experimental designs, experimental design choices may also be made *in preparation* for *fitting* joint models. The data from some experimental designs are more easily modelled due to (1) larger bases of research knowledge for some specific theories of human cognition and brain signals and (2) the availability of algorithms and software packages that allow fitting certain classes of models and data. That is not to say that some experimental and modeling work should not occur, only that more theoretical development, technical expertise, and/or model-fitting algorithm development is needed to answer some questions. Note that the idea of choosing experiments based on current states of theoretical knowledge and algorithm availability may be somewhat distasteful to researchers who feel that theory and algorithm development should occur to explain any collected data. However, each of these two intermediate steps likely require extensive research (e.g., see Guest & Martin, 2021, for a discussion on theory development).

Discrete choice and response time data resulting from two-alternative forced-choice (2AFC) experimental tasks, or similar tasks, are known to be easily studied using signal detection theory and sequential sampling models. However, work on developing models of more complex decisions, such as choices in continuous space, is still somewhat new (Smith, 2016; Ratcliff, 2018; Kvam, Marley, & Heathcote, 2023). And while there is a large body of work on developing models for describing choices and response times for more than two alternatives (Busemeyer, Gluth, Rieskamp, & Turner, 2019; van Ravenzwaaij, Brown, Marley, & Heathcote, 2020; Thomas, Molter, & Krajbich, 2021; Krajbich & Rangel, 2011; Hawkins & Heathcote, 2021; Heathcote & Matzke, 2022), models of multi-alternative forced choice (MAFC) tasks have not been as widely utilized as models of 2AFC tasks in studies testing the influence of experimental manipulations on parameters (e.g., Dutilh et al., 2019) nor in studies of stable measurements of individual differences (e.g., Schubert, Frischkorn, Hagemann, & Voss, 2016; Lerche et al., 2020), with some notable exceptions (e.g., Rouder, Province, Morey, Gomez, & Heathcote, 2015). If a researcher only cares about a scientific question that does not depend on the specific type of choice, then the researcher might choose the 2AFC task because of the current state of knowledge. Another concern is that researchers may find model fitting limited to existing packages which lack procedures for fitting MAFC and continuous choice tasks. However, in the future, it is likely that MAFC model-fitting procedures will be further rigorously tested and more widely applied, with new packages being released (e.g., Stevenson, Innes, Boag, & Heathcote, 2023; Villarreal et al., 2023). We also expect that model-fitting procedures will become more flexible in their implementation (e.g., see Radev, Mertens, Voss, Ardizzone, & Köthe, 2020). Furthermore, if the goal is to develop new methods and/or to test something specific about multiple alternatives or choices on a continuous scale, then a MAFC or continuous choice experimental task would be best.

Some M/EEG signals will be more easily found (e.g., differentiated from other signals) in certain experimental conditions due to prior knowledge from a wealth of literature about these signals. For this reason, the same experimental tasks are used often in M/EEG research and electrophysiology across experiments, such as the random dot motion (RDM) task (e.g., see Newsome & Pare, 1988; Gherman & Philiastides, 2018). For instance, the centro-parietal positivity (CPP) is known to occur in tasks where a visual or auditory stimulus ramps up or down in signal intensity, including RDM tasks (O’Connell et al., 2012; Kelly & O’Connell, 2013; Rangelov & Mattingley, 2020). Combining all these experimental considerations, the best visual stimulus to test a simple hypothesis of the relationship of CPP to an evidence accumulation rate during decision-making might be a RDM where the participant must differentiate either leftward or rightward motion during each trial (i.e., a 2AFC task). Note that researchers should not be limited to prior research, and researchers should feel free to design new experimental tasks and new experimental designs. Diversity of experimental design ideas will always remain important for the growth of the field of joint modeling and the growth of knowledge.

## Collection and preprocessing of M/EEG for joint modeling

### Software for processing of M/EEG data

The software for both preprocessing and analysis of M/EEG data are either stand-alone programs or based on either the MATLAB (The MathWorks Inc., 2022) or Python programming languages. This is due to a (likely self-reinforcing) preference of cognitive neuroscientists generally for MATLAB and Python over R. EEG analysis packages exist for R, but are being still developed and not as widely used, such as eegUtils (Craddock, 2023). We recommend using established toolboxes, such as MNE (Gramfort et al., 2013) in Python, or FieldTrip (Oostenveld, Fries, Maris, & Schoffelen, 2011) or EEGLAB (Delorme & Makeig, 2004) in MATLAB. Tutorials and many walk-through example analyses are readily available online for all three toolboxes. In this section, we introduce readers to general principles about EEG analysis. We encourage readers to follow the *Further readings* section and consult specific online toolbox manuals if beginning EEG analysis for the first time.

### Understanding artifactual processes in M/EEG data

M/EEG contains many overlapping sources of information, including brain-generated sources and artifactual sources (Nunez & Srinivasan, 2006; Nunez, Nunez, & Srinivasan, 2016; Fitzgibbon et al., 2016). Due to the complexity and the size of information in collected M/EEG, parameter estimation of joint models of behavior and M/EEG usually requires extraction of specific M/EEG signals. One important step in this process is the removal of artifact, specifically muscle-generated electromyographic (EMG) signals, environmental electrical artifacts, and physical movement artifacts that are prevalent in scalp-recorded EEG recordings (Whitham et al., 2007; Nunez et al., 2016). Unless those artifacts are removed, or specifically accounted for in a joint model, these sources of artifact will likely add noise that affects results of joint modeling. These artifacts could thus greatly influence the results of neurocognitive models in parameter estimates or the results of model comparisons (e.g., see Hawkins, Mittner, Forstmann, & Heathcote, 2017).

**Fig. 3:**
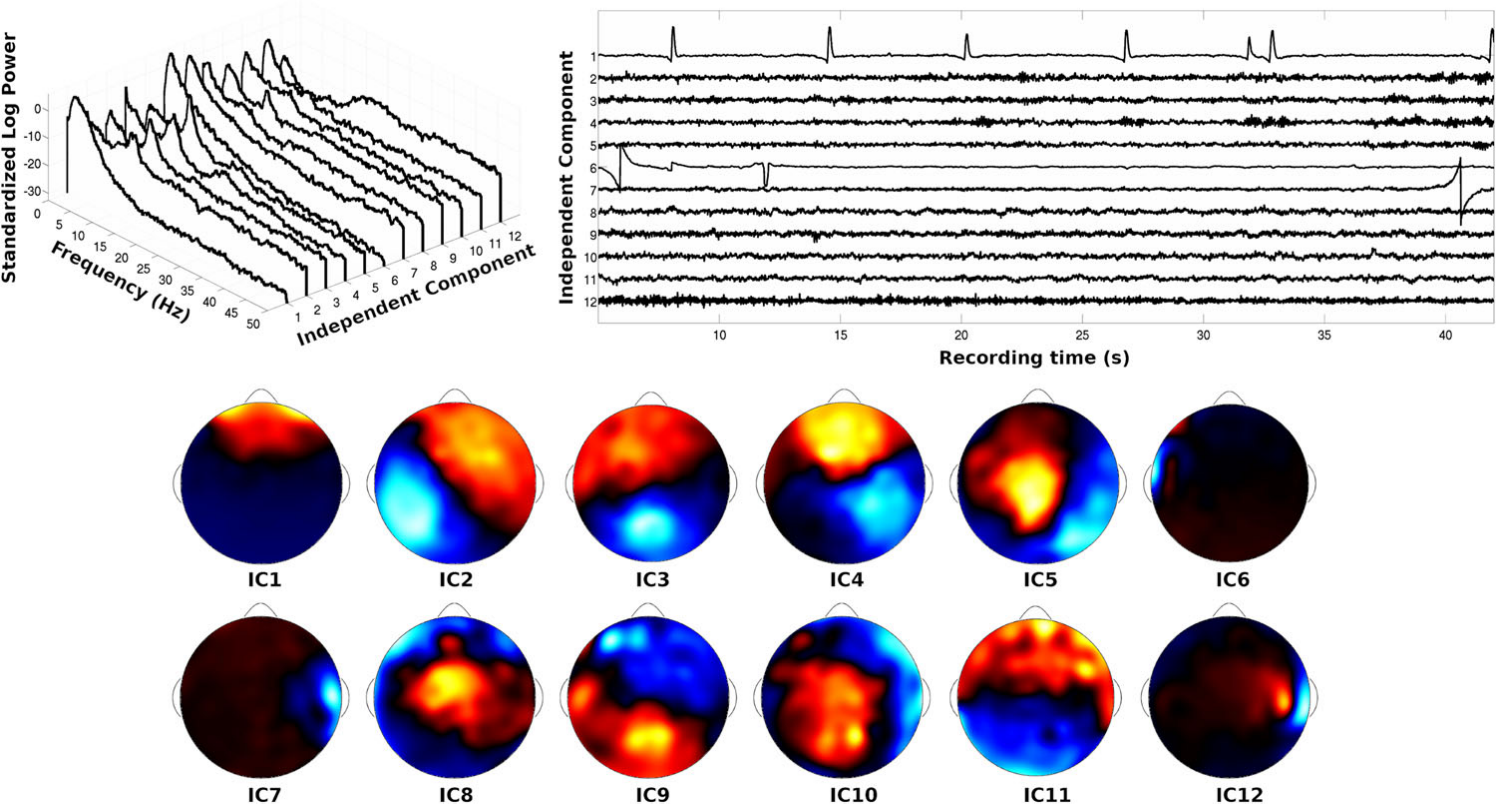
(*From top-left, clockwise*) Power spectra, time courses, and spline-interpolated channel weights from the first 12 independent components (ICs). The independent component analysis (ICA) algorithm was performed on an EEG record in which a participant was fixating on a computer monitor. ICs that are likely to reflect artifacts can be subtracted from the EEG data before neurocognitive modeling. IC1 is indicative of an eye blink. IC6 and IC7 are indicative of temporary changes in channel impedances. IC12 is indicative of some muscle artifact. Note this figure is adapted with permission from Fig. 4 by Nunez et al. (2016)

Electrical and movement artifacts in scalp-recorded EEG records can be reduced by proper recording practices. Usually the goal of these practices is to (1) keep consistent electrical contact of the ground and reference electrodes to the scalp, (2) keep consistent electrical contact of all other electrodes (or to remove those electrodes from later analysis, if some electrodes can be removed without much loss of information, such as in high-density EEG), and (3) by removing or shielding external sources of electrical artifact from the area of recording. Electrodes with higher impedances typically have larger amplitude environmental noise (with large noise amplitudes at 60 Hz or 50 Hz depending upon the place in the world in which the recording takes place) that can be removed with online or offline targeted filtering (Kappenman & Luck, 2010). However, also large impedances can be indicative of an electrode that is not making consistent electrical contact with the scalp and could produce high amplitude movement artifact. Inconsistent contact of an electrode could lead to sudden changes in impedance that can affect EEG records of that electrode. Most commercially available EEG systems have easy-to-implement methods of measuring electrode impedance. Those electrodes that have very high impedances can be given special attention (using system recommended cutoffs depending upon the EEG cap and amplifier), such as making sure that the electrode is making stable contact with the skin of the scalp through hair. Unless using “dry” EEG systems, an appropriate amount of conductive gel or saline is also important to maintain electrical contact. However, it is also important not to use too much conductive solution to avoid electrical bridging (Greischar et al., 2004). We recommend reading some of the existing recommendations from various EEG laboratories resources for discussions of best practices for EEG data collection (e.g., Farrens, Simmons, Luck, & Kappenman, 2020; Boudewyn et al., 2023), as well as recent work on improving recording practices for different hair types (e.g., Etienne et al., 2020).

In addition to proper recording practices, additional artifact correction is almost always performed on EEG data, especially for eye blinks, EMG, cardiovascular-generated electrocardiographic (EKG) signals, and miscellaneous movement artifacts. For joint modeling, this artifact correction will usually be performed offline, as opposed to BCI applications where artifact correction is performed online. Artifact correction is also helpful for magnetoencephalographic (MEG) and intracranial electroencephalographic (iEEG) records, although the prevalence of different types of artifacts will differ across modalities and recording systems. Techniques to mitigate artifacts before parameter estimation is especially necessary due to the simple assumptions often made intrinsically in many joint models of M/EEG and behavior. Because brain-generated EEG recorded from the scalp will not manifest as sudden large amplitude spikes after filtering by the skull and skin, common offline artifact correction techniques include removing epochs of data or specific electrode records that surpass a particular amplitude cutoff are useful and basic artifact corrections.

Another popular method that deserves special consideration is independent component analysis (ICA Makeig, Bell, Jung, & Sejnowski, 1996) for scalp-recorded EEG and MEG. ICA finds linear mixtures across M/EEG channels that are statistically independent as possible by finding maximal non-Gaussian mixtures. The resulting components have M/EEG-like times courses where, if the time dependency were ignored and the time samples were randomly shuffled, the resulting distributions would have minimum mutual information or be separated on the basis of kurtosis (Hyvärinen & Oja, 1997; Jung et al., 2000). In practice, these methods often yield non-normal mixtures that have distributions with outliers, and thus ICA algorithms are especially good at extracting certain artifacts within multiple EEG channels such as eye blinks (IC1 in Fig. 3), eye movements, EKG, and temporary changes in electrode impedances (IC6 and IC7 in Fig. 3). Sometimes ICA algorithms can also find some EMG artifacts that can be easily identified in the EEG data (IC12 in Fig. 3). Often ICs are identified manually through visual inspection. However, recent algorithms have sought to remove some of the subjectivity in IC identification, with specific algorithms (e.g., see Mognon, Jovicich, Bruzzone, & Buiatti, 2011) or with Artificial Neural Networks trained on may expert evaluations (Pion-Tonachini, Kreutz-Delgado, & Makeig, 2019; Li, Feitelberg, Saini, Höchenberger, & Scheltienne, 2022) – see the first *Exercise* for additional help on this topic. These components can then be extracted from multiple electrodes, and the resulting EEG data can be converted back to channel space or be kept in component space for joint modeling. EEG components (weighted mixtures of electrodes) are discussed in detail below.

Note that it is unlikely that all artifact will be removed using these methods, especially EMG artifact in scalp-recorded EEG (Whitham et al., 2007; Nunez et al., 2016). So to further avoid artifact in scalp-EEG, it is best to choose specific methods of signal extraction. This is typically performed outside of joint modeling, but future researchers may be able to model these methods explicitly to retain sources of noise in the model. These methods include popular EEG-band limited analyses (e.g., calculating 8 to 13 Hz alpha power over posterior electrodes in Ghaderi-Kangavari et al., 2023a) and event-related potential (ERP) analyses that mitigate artifactual components through averaging across trials (e.g., N200 latencies in Nunez et al., 2019a).

Finally, M/EEG outliers could drive the entire modeling results. A choice must be made between explicit removal and explicit modeling of lapse and artifactual processes. Note that there will be differences between artifactual (non-brain generated M/EEG) and brain generated M/EEG that is related to *some* cognitive process but not related to the cognitive process of interest. A true neurocognitive reason for an outlier could be, for instance, because the participant is mind wandering during that trial (e.g., see Hawkins, Mittner, Forstmann, & Heathcote, 2022). It is a question for new joint modeling work whether inclusion of non-brain generated M/EEG artifactual processes and/or mixtures of cognitive processes within the joint modeling itself would be beneficial to understand the researchers’ specific hypotheses and questions.

### Extraction of relevant M/EEG signals

In previous research, most researchers have extracted specific EEG signals before joint modeling (e.g., Frank et al.,^2015^; Nunez et al., 2017; Nunez et al., 2019a). Targeted extraction of these specific EEG signals can be especially beneficial for joint modeling, particularly when those signals have a rich literature of prior research and cognitive theory. We will concentrate here on popular EEG signals studied within cognitive neuroscience.

Event-related potentials (ERPs) are defined as averages of M/EEG across experimental trials, time-locked to specific events such as the onset of a visual stimulus (a visual evoked potential; VEP) or the execution of a response such as a button press (a motor evoked potential; MEP). ERPs can be computed by simply averaging the M/EEG signals *v* over *N* trials *i* such that resulting signal $\mu$ varies over a time index *t*, time-locked to an experimental event:

9 $$\begin{aligned} \mu _{t} = \frac{1}{N}\sum _{i=1}^{N} v_{ti} \end{aligned}$$

ERPs also have rich literature (Luck, 2012, 2022), from which best practices can be recommended. This literature can also be used to generate new confirmatory and exploratory hypotheses that could be answered with joint modeling methods. After calculation of ERPs using the equation above, specific peak latencies, amplitudes, or deviation times from baseline are typically extracted, either positive or negative peaks. Common ERPs are the negative N200 peak approximately 200 ms after the onset of a visual stimulus in occipital and parietal electrodes (sometimes labeled N1 for the first negative peak), recently thought to encode the onset of evidence accumulation during decision-making (Nunez et al., 2019a). Another common ERP is the positive P300 peak at least 300 ms after the onset of a visual stimulus; this ERP is also called the cento-parietal positivity (CPP) during specific decision-making tasks (Twomey et al., 2015), discussed extensively earlier and used in example Models 2 and 3. Common MEPs are the readiness potential (RP) and the related lateralized readiness potential (LRP) (Gluth et al., 2013; Lui et al., 2021, e.g.). ERPs can also be estimated on single-trials (e.g., Nunez et al., 2017; Bridwell et al., 2018; Nunez et al., 2019a).

Frequency or time-frequency decompositions, such as Fourier and wavelet analyses, are also common methods used to extract specific signals in M/EEG (Cohen, 2014). These decompositions form the basis of derived measures, such as M/EEG coherence, and typically rely on signal-to-noise ratios. Frequency and time-frequency measures have been used in neurocognitive modeling of decision-making (Frank et al., 2015; Polanía et al., 2014) and can be event-locked or stem from endogenous rhythms not related to the timing of the task. Many algorithms in high-level programming languages such as the fast-Fourier transform are sufficient to estimate these signals, although best practices in EEG and standard EEG conventions should be known (Nunez et al., 2016). Alternatively, there are other algorithms developed specifically to extract specific band-limited waveforms, such as finding 80–250-Hz high-frequency oscillations (HFOs) that last only for a few milliseconds in iEEG data (Charupanit & Lopour, 2017; Nunez, Charupanit, Sen-Gupta, Lopour, & Lin, 2022). One warning for scalp EEG is that high frequencies (approx. $> 20$ Hz) typically contain more EMG artifact (Whitham et al., 2007), so care must be taken when interpreting the results of measures derived from high frequencies embedded in joint models. Although MEG systems may be more robust to EMG artifact (Claus, Velis, Lopes da Silva, Viergever, & Kalitzin, 2012; Muthukumaraswamy, 2013), and newer MEG systems could be even more resistant to EMG artifact (Ilmoniemi & Sarvas, 2019). Another caution is that researchers have shown heterogeneity in the power bands across and within participants (Nunez, Wingeier, & Silberstein, 2001), as well as heterogeneity in the waveforms themselves (Donoghue, Schaworonkow, & Voytek, 2022). Therefore, care must be taken when extracting specific signals for joint modeling.

Steady-state evoked potentials (SSEPs), and in particular steady-state visual evoked potentials (SSVEPs) and related steady-state auditory potentials (SSAEPs), are a special case of band-limited analysis where the frequency band of interest in the M/EEG results from a processing stimuli at a certain presentation rates or “flicker” (Regan, 1977). For instance, a Gabor patch flickering at 15 Hz will result in a large, narrow-band, 15-Hz response (and often harmonics of 15 Hz) in EEG. This is the result of the cortex receiving and processing signals at this rate, which is expected of a linear system. Some researchers have found evidence that endogenous EEG signals may also “entrain” to the stimulus frequencies (Srinivasan, 2004; Ding, Sperling, & Srinivasan, 2006). SSEP analyses could be particularly useful for fitting joint models because the amplitude or phase-locking across trials is thought to index within individual and individual differences in attention (Ding et al., 2006). We have previously explored how individual differences in attention as measured by SSVEPs affected cognitive components of decision-making (Nunez et al., 2015).

Typically ERPs, SSEPs, and power in different (time-)frequency bands, are observed in replicable scalp and brain locations. These specific EEG signals often have consistent cognitive interpretations found in the cognitive neuroscience literature, e.g., see Table 1. However, the exact electrode/sensor locations will differ from participant to participant, and could even change within a participant due to varying artifactual sources and electrical contact of the electrodes over the course of a long experiment. Furthermore, within the field of model-based cognitive neuroscience modeling, there is a need to better utilize overlapping information in M/EEG data to improve descriptions of cognitive theory (see Borst & Anderson, 2015; Bridwell et al., 2018; Weindel, van Maanen, & Borst, 2023). For intracranial EEG (iEEG), spatial filters can also be useful in extracting relevant EEG features for joint modeling (e.g., see Schaworonkow & Voytek, 2021). Therefore, weighted averages across channels should also be considered. We will refer to these sets of analyses as “component analyses” for finding mixtures, typically linear mixtures, of M/EEG data that may better reflect the underlying source components present on the scalp or in intracranial electrode space (Parra, Spence, Gerson, & Sajda, 2005). Component analyses will extract a weighted average of electrodes/sensors to improve the signal-to-noise ratio of mixtures. Previously mentioned independent component analysis (ICA) is one example algorithm, but other methods like principal component analysis (PCA) (e.g., Nunez et al., 2017; Nunez et al., 2019a), canonical correlation analysis (CCA) (e.g., van Vugt, Simen, Nystrom, Holmes, & Cohen, 2012), and explicitly modeling mixtures over electrodes/sensors in joint models can also be considered.

Preprocessing of M/EEG may be theoretically undesirable since extracting specific signals often involves removing potentially useful information from the M/EEG signals, which could be better accounted for with statistical models in joint modeling. In all our previously published work (Nunez et al., 2015, 2017, 2019a; Lui et al., 2021), we extracted specific EEG potentials before joint modeling. While these methods are useful for testing specific theories of visual attention, motor processing, or visual encoding, they are not as suitable for understanding parallel processes that occur during decision-making. The future of joint modeling techniques should better account for full M/EEG data to improve prediction and hypothesis testing. This can be achieved by embedding mixture models of M/EEG signals, either based on brain connectivity and neural network behavior (with a model of electric volume conduction to the scalp, see paper by Nunez, Nunez, & Srinivasan, 2019b) or through more non-parametric methods based on mixtures of oscillating signals.

## Implementing model-fitting procedures and estimating parameters

Finding parameter estimates from a proposed model can be difficult. There are many more restrictions on parameter fitting than model simulation (due to difficulty in maximizing *likelihood* spaces or sampling from *posterior distributions*). Many joint models will also not be *identifiable*, as discussed below. However, multiple free programs exist to help you fit joint models of M/EEG and behavior. Most require some knowledge of a programming language and sampling methods.

### Terms in model fitting

One term that is often used in model *fitting* is that of the *likelihood*. Each distributional statement represented with the $\sim$ symbol in this paper has an associated equation that describes the probability of taking certain data values when calculating the *integral* of the equation over a range of possible values. Thus, the integral of the function over all possible parameters is always 1, indicating that the data being somewhere in the entire possible range has probability $=1$. This equation, the *probability density function* (pdf) is called the *likelihood function* *L* whenever it is considered to vary over parameters for observed data. The integral of this function *L* now no longer represents probability (and the function has a different shape), because the function varies over a range of parameters and was not defined to represent probability in this way.

For instance, if we want to estimate the standard deviation $\sigma$ of raw EEG data in one electrode *e* over a certain period of time. We could assume that *e* is distributed normally with mean 0 and an unknown standard deviation $\sigma$. This is given by the distributional statement $e_{s} \sim Normal(0, \ \sigma ^{2})$ for each sample *s*. If we have 1024 samples of EEG $e_{s}$, say 1 second of recording with an amplifier that has a sample rate of 1024 Hz, then we could find the *maximum likelihood estimator* by placing those exact data values $\textbf{e}$ (bold indicating a vector of data samples $e_{s}$) in a joint pdf, given by individual normal pdfs for each EEG sample multiplied together:

10 $$\begin{aligned} f(\sigma , \textbf{e}) = \prod _{s=1}^{1024} \frac{1}{\sigma \sqrt{2\pi }} \exp \left( -\frac{e_s^2}{2\sigma ^2}\right) \equiv L(\sigma | \textbf{e}) \end{aligned}$$

Note that even though the likelihood function *L* for $\sigma$ is derived from and has the same mathematical equation, the shape of this function is not a bell curve over certain values of $\sigma$ for now known data values $\textbf{e}$ because it is now a function of parameters and not data. With this likelihood function *L*, we can find a value for $\sigma$ that describes the maximum likelihood by finding the value of $\sigma$ at the peak *L* value (e.g., by differentiating the equation and setting it equal to zero). This results in a good *estimate* of the true, never observed, EEG standard deviation. Note that, confusingly, this *estimate* is often called the “standard deviation of the data” in conversation. The likelihood function is also used in Bayesian inference, where the shape of the *posterior distribution* over possible parameter values is given by the shape of the likelihood function *L* multiplied by the shape of the *prior* distribution.

Most programs to fit joint models have predefined likelihood functions, and only under special conditions would you need to write your own likelihood function. This is true in all *probabilistic programming languages* (see below) as well as most other existing programs written to fit models. Readers who want additional information, are confused by these concepts, or who want to define their own likelihood functions should consult chapters 4 and 6 of the textbook by Farrell and Lewandowsky (2018) and see additional *Further readings* for this section – found at the end of this paper.

### Avoiding model complexity

To test specific hypotheses or compare theories, a perfect explanation of all relevant M/EEG signals and behavioral data is often unnecessary and could result in overfitting the model (see Navarro, 2019). The degree of complexity needed in the fitted model(s) will depend on the goals of the researcher. In DDMs, choosing between two discrete choices is assumed to occur due to particular time-varying sampling of relative evidence. The rate of sampling of evidence is assumed to change both during a single choice due to within-trial variability in a random walk process, but also change across many similar choices, due to trial-to-trial variability (Ratcliff et al., 2016). In an experiment with the participant making a choice during every experimental trial, expected across-trial changes in the parameters are often modeled with across-trial variability parameters. While it is expected that humans do have variability in strategy, attention, and response cautiousness, etc. across trials, across-trial variability parameters of a full DDM model cannot be easily estimated with behavior alone (Boehm et al., 2018). This problem of parameter estimation can be at least slightly improved with the addition of EEG signals on single trials (Nunez et al., 2017; Hawkins et al., 2017). Therefore, when fitting behavioral data to joint models, we often make the choice to fit simple DDM without any across-trial variability in the DDM parameters that is not described by the single-trial EEG measures (Nunez et al., 2017, 2019a). Other joint modeling research has often included across-trial variability in evidence accumulation rates but no other parameters (e.g., Frank et al., 2015; Ghaderi-Kangavari et al., 2022). In past work, we have purposely not included some across-trial variability parameters because we have shown in simulations that the question of interest about the M/EEG-cognitive relationship can be answered without more complex models, knowing that the greater mathematical theory of decision-making does have variability and that the data would better be described by more complicated models. However, if we were differentiating between models that are very similar in their predictions, we might want to include trial-to-trial variability parameters or more precise M/EEG correlates of evidence accumulation.

**Fig. 4:**
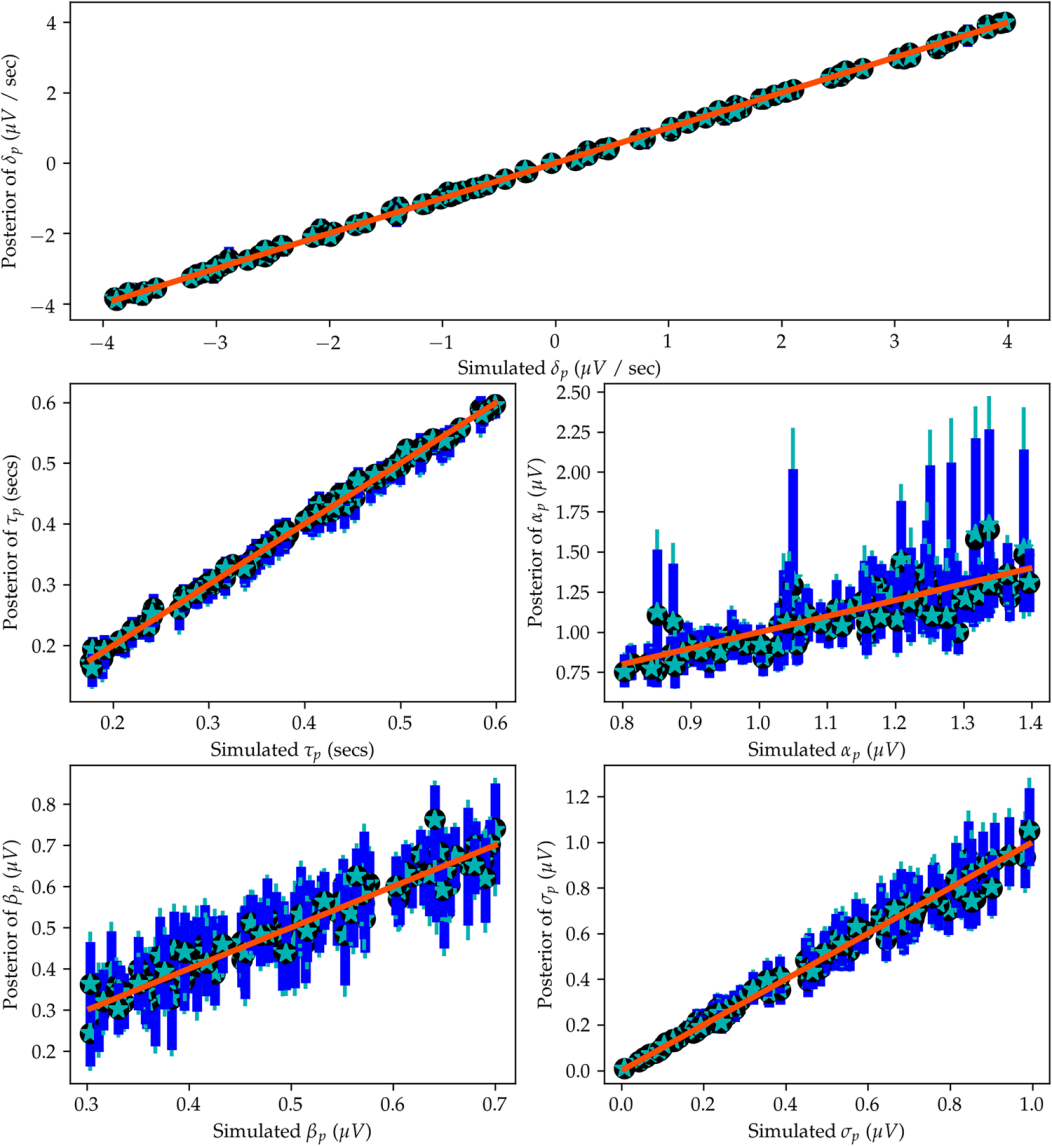
The recovery of five parameters from Model 3. We simulated Model 3 using the provided Python code with 100 simulated participants and 100 trials for each participant. The *x*-axis of each plot is the true simulated parameter and the *y*-axis is a summary of posterior samples. The mean of the posterior distributions is given by *teal star symbols* and the median of the posterior distributions is given by *black circles*. Uncertainty about each parameter estimate is given by the $95\%$ credible intervals of *dark blue lines*, and $99\%$ credible intervals of *teal lines*

### Parameter recovery of simulated models

Simulation is especially important for newer models and model-fitting procedures that are not widely used. Because jointly fitting neural data and human behavior is not widely used within cognitive neuroscience and psychology, the majority of model-fitting procedures that readers may implement will fall into this category. Thus, researchers interested in neurocognitive models should always simulate and refit new models to understand parameter recovery. The parameter estimates from new models and new model-fitting procedures should always be verified by refitting data from simulations before modeling results can be trusted to be self-consistent, whether or not the model is reflective of reality or a specific hypothesis. For instance, model-fitting procedures could give realistic results, but fail to recover the same parameters when simulated. This means that the parameter estimates recovered from model-fitting will not reflect reality, even if the simplified model is completely true. In addition, some parameters of a model may recover, and therefore be relevant to analyze in real data, while other parameters of a model will not recover. In Fig. 4, we show recovery of the five of six parameters from Model 3 when using Markov chain Monte Carlo (MCMC) sampling in JAGS (discussed below) with the JAGS Wiener plugin (Wabersich & Vandekerckhove, 2013). We fit a model that assumes trial-to-trial variability in CPP slopes and not drift-rate (e.g., assuming $\eta$ = 0). The code for this simulation, parameter recovery, and plot is available at https://github.com/mdnunez/pyhddmjags/blob/master/simpleCPP_test.py as of March 2022, along with a shortened annotated version in the *Appendix*.

### Comparing models

Model comparison is useful when multiple theories could describe the data and the evaluation of hypotheses would depend on the theory assumed. Model comparison can also be used to evaluate competing theories directly. Therefore, model comparison in joint cognitive modeling of M/EEG and human behavior is almost always beneficial. Model comparison can be based on multiple dimensions, but typically researchers are interested in the models that provide the most predictive and/or realistic accounts of the neural and behavior data. Model comparison could therefore be based on how well the model predicts data or how well models evaluate the hypothesis versus an alternative hypothesis. For example, if we were interested in the hypothesis that the CPP reflects evidence accumulation *exactly*, we could compare a model where the slope of the CPP describes the evidence accumulation rate itself, e.g., Model 3, to a more general model where the relationship between the CPP and evidence accumulation rate can be any value (see Exercises). If Model 3 describes the data nearly as well as the more generalized model, then we have evidence for the hypothesis that the CPP reflects evidence accumulation exactly.

How well a model *predicts* data used to fit the model itself is often used as a measure of performance (Blohm et al., 2020). This is called *in-sample* prediction. However, evaluating models based only on in-sample prediction can result in *overfitting* the data. Overfitting describes the situation in which additional in-sample prediction is gained through model complexity that is unrelated to the underlying true generative process, resulting in worse *out-of-sample* prediction. Out-of-sample prediction refers to how well a model predicts data that it was not fit to. We refer readers to a discussion about this topic by Aki Vehtari at https://avehtari.github.io/modelselection/CV-FAQ.html (Vehtari, 2023). Out-of-sample could also refer, although this is not traditionally in the definition, to how well it generalizes to similar data from other experiments (see Busemeyer & Wang, 2000). Note that prediction of in-sample and out-of-sample data can be used to compare models that differentiate specific hypotheses, but it is often not necessarily to perfectly describe or predict EEG and/or behavioral data due to the presence of artifacts and noise in EEG and contaminate behavioral data not related to the cognition of interest.

For evaluating neurocognitive models of M/EEG and human behavior, we prefer out-of-sample prediction (e.g., Nunez et al., 2017; Schubert et al., 2019). Out-of-sample prediction typically involves taking at least one subset of the data out before fitting the model to the remaining “in-sample” data. For instance, one could split a data set where $80\%$ of the data is used to fit the model and $20\%$ of the data is used to evaluate out-of-sample prediction. One method to evaluate the similarity of predicted data to the actual data is with a *proportion of variance explained* calculation. For instance, we have previously calculated $R^{2}_{\text {pred}}$ of participants’ accuracy and correct response time 25th percentiles, medians, and 75th percentiles (Nunez et al., 2015, 2017). $R^{2}_{\text {pred}}$ is a measure of percentage variance in a statistic *T* (e.g., accuracy, correct-RT median, etc.) explained by in-sample or out-of-sample prediction. It is a function of the mean squared error of prediction (MSEP) and the sample variance of the statistic *T* based on a sample size *J* of data observations. $R^{2}_{\text {pred}}$ is defined as:

11 $$\begin{aligned} R^{2}_{\text {pred}} = 1-\frac{\sum ^{J}_{j=1}(T_{j}-T_{(\text {pred})j})^{2}/(J-1)}{\sum ^{J}_{j=1}(T_{j}-\bar{T})^{2}/(J-1)} = 1 - \frac{\text {MSEP}_{T}}{\widehat{\text {Var}[T]}} \end{aligned}$$

As one example, we could compare the out-of-sample prediction of Model 2A to Model 2B that was fit to $80\%$ of the data from Experiment 1. The observations for each statistic T in the $R^{2}_{\text {pred}}$ equation would be for every participant *j* with sample size *J* being the number of participants.

There are other methods to evaluate out-of-sample prediction, such as calculating the log-likelihood under predicted data (e.g., see Figure 9 of Turner, Rodriguez, Norcia, McClure, & Steyvers, 2016). Models’ prediction ability can also be evaluated with plots such as quantile–quantile (Q–Q) plots of measured versus predicted data quantiles. Because we typically use Bayesian methods, we generate posterior predictive distributions for in-sample and out-of-sample data. We can then create posterior predictive coverage plots (e.g., see Supplementary Materials of Nunez et al., 2017) or Q-Q plots. Plotting often provides additional information to more quantifiable measures such as $R^{2}_{\text {pred}}$ or a similar measure.

Cross-validation refers to methods where out-of-sample prediction is performed repetitively on different subsets of data (e.g., a new $20\%$ of the same data set iteratively). Because cross-validation lowers the impact of outliers in the out-sample, cross validation can be useful for modeling M/EEG data because the presence of outliers is commonplace. However, care must be taken to avoid changing parameters of the model or model-fitting procedure based on the results of cross validation because this would make the cross validation process less reflective of the true predictive ability of the model.

If out-of-sample prediction is not available, then often penalizing by model complexity after in-sample prediction is used. This is often why information criteria measures are used (e.g., Ghaderi-Kangavari et al., 2023a; Ghaderi-Kangavari et al., 2022). Essentially, these measures are in-sample prediction measures that penalize for model complexity. Akaike information criterion (AIC), Bayesian information criteria (BIC), deviance information criteria (DIC), and re-weighted variations of these measures are often used. However, these measures may yield different results. For instance, it is thought that BIC more often favors models that match the ground truth while AIC more often favors models that are predictive of new data (Aho, Derryberry, & Peterson, 2014; Chandrasekaran & Hawkins, 2019) Therefore, it is important to pick one ahead of time and stick to it, or preregister the modeling analysis (Lee et al., 2019; Vandekerckhove et al., 2019).

Simulation is also important if you wish to perform model comparison. Some models may fit data better not because the underlying theory is a better reflection of reality, but because the models capture some contaminant process better. That is, neither model is correct, but the worse-fitting model is a better description of reality. Simulations of multiple models with contaminant processes before performing parameter recovery for each model can thus reveal which model and model-fitting procedure best recover true parameters. Simulation is also important for comparing models that predict both EEG and behavioral data, such as Model 3, to other such models or models that only predict one data type. It is not immediately clear whether M/EEG or behavior should be favored when evaluating in-sample and out-of-sample prediction to compare models, and predictions of this data could change with each new model. Therefore, simulations should be performed to make sure the parameters of interest are recovered when comparing models, as well as understand how model changes affect predictions of multiple data types. For an example of joint modeling for EEG and behavior, see work by Ghaderi-Kangavari, Rad, and Nunez, (2023b).

We separate here confirmatory research from exploratory research. When discovering the influence of EEG measures on parameters that describe decision-making behavior, it is beneficial to explore various model types that may better match the theoretical evidence accumulation process. This can be achieved, for instance, either by fitting parameters from different sequential sampling models (SSMs) directly (preferred) or simulating a variety of SSMs and then exploring how similar parameters can be recovered in other models. Confirmatory research, on the other hand, necessitates large samples, pre-deciding an analysis plan that includes the specific joint model to test (and perhaps preregistration of that analysis plan and model), and requiring strict standards for hypothesis acceptance, such as in clinical trials (Lee et al., 2019). However, it is worth nothing that there exists a spectrum between exploratory and confirmatory research that has been discussed elsewhere (e.g., Devezer, Navarro, Vandekerckhove, & Ozge Buzbas, 2021).

### Fitting complex models using Bayesian methods

We generally prefer to use programs that use Bayesian Markov chain Monte Carlo (MCMC) sampling and allow a large amount of flexibility to change the model structure. Probabilistic programming languages such as JAGS (Plummer, 2003), Stan (Carpenter et al., 2017), and PyMC3/4 (Salvatier, Wiecki, & Fonnesbeck, 2016) all make this process incredibly easy by allowing you to write your own complex models, but without needing to write your own samplers. Popular wrapper programs have also been created around these programs that make fitting certain models even easier (Wiecki, Sofer, & Frank, 2013; Bürkner, 2017), and there are other easy-to-use programs that implement their own samplers (Heathcote et al., 2019; Stevenson et al., 2023). Note that Bayesian analysis can be quite easy to learn for those that have a background in some mathematics and statistics. A nice introduction to Bayesian analysis is given by Etz and Vandekerckhove (2018). See also books by McElreath (2020) and Gelman et al. (2014).

All the aforementioned programs also allow the modeler to easily implement hierarchical parameters which can better account for variance across experimental conditions, participants, sessions, etc. Hierarchical parameters can often better account for data with multiple modes of data (Lee, 2011; Turner et al., 2016), such as EEG and human choice response times. Hierarchical models often yield better estimates of parameters due to “shrinkage” towards the mean parameters rather than fitting a model per participant or experimental condition, which could lead to overfitting and misestimation (see Chapter 5 of Gelman et al., 2014). Examples for fitting behavioral DDMs and neurocognitive DDMs using Python, JAGS, and Stan, with the models themselves written in JAGS and Stan code, are given in the repository https://github.com/mdnunez/pyhddmjags. We encourage readers to run the example models in this repository if they are interested in using JAGS and Stan with Python. Note that connectors to JAGS and Stan also exist in R (for examples, see https://github.com/kiante-fernandez/Rhddmjags) and other programming languages.

The programmatic implementation to generate parameter estimates from joint models that we preferred in the past is JAGS (Plummer, 2003). JAGS is now a somewhat older program, but nicely contains multiple MCMC samplers and chooses among them based on the user-defined model. Custom distributions can also be added to JAGS (Wabersich & Vandekerckhove, 2014; Villarreal et al., 2023). JAGS uses Bayesian MCMC samplers to fit models to data and can easily fit joint models to multiple data types. For instance, we fit a simplified version of Model 3 in JAGS with $12,\!000$ original samples in each of six chains for each parameter (see https://github.com/mdnunez/pyhddmjags/blob/master/simpleCPP_test.py). After removing the first 2000 samples as a “warm-up” or “burn-in” and then keeping only every 10th sample, i.e., using a “thinning” parameter of 10, this results in 1000 posterior samples in each chain for 1000 * 6 = 6000 samples from the estimated posterior distributions for each parameter.

To assess whether the model is reaching a unique solution (i.e., unique joint posterior distributions), we can both inspect our MCMC chains but also gauge certain model convergence diagnostics (Gelman et al., 2014). The Gelman–Rubin statistic and the number of effective samples are calculated (Gelman et al., 2014). The Gelman–Rubin statistic assesses the convergence of MCMC samplers by comparing the between-chain variance to the within-chain variance of each parameter, with Gelman–Rubin statistics $\le 1.1$ thought to be a necessity for convergence. We also implemented the recommendation by Gelman et al. (2014) (see footnote in the 3rd Edition on page 283) to split the chains in half before calculating the Gelman-Rubin statistic in order to account for non-stationary chains. The “effective number of samples” equation scales the total sample number for each parameter posterior by autocorrelation in the chains in order to estimate an independent number of samples. Larger effective samples for each parameter in the model are better. The chains for parameters with the largest Gelman–Rubin statistics and smallest effective number of samples are also visually inspected to ensure convergence. In publications we typically report the maximum Gelman–Rubin statistics across all parameters, and we have recently started to report the minimum number of effective samples across all parameters.

### Prior distributions in Bayesian models

When using Bayesian methods we often must choose prior distributions of parameters, this is also true for estimating joint models of M/EEG and behavior with Bayesian methods. When possible, we pick prior distributions based on previous publications, and such that the prior distributions have weight over plausible values of the parameters. For instance, random draws from a normal distribution with a mean of .5 and a standard deviation of .25 will result in $68.2\%$ of those draws within .25 and .75, and $95.4\%$ of those draws within 0 and 1. [0, 1] is the domain of the relative start point parameter $\beta$ that encodes initial evidence bias in a DDM. Therefore, a normal distribution with mean .5 and standard deviation of .25 truncated to the domain [0, 1] would be a good prior distribution for this parameter, disregarding algorithmic reasons why we might pick different priors (such as in true Gibbs sampling).

There is ongoing research about what the best prior distributions are, depending upon the type of sampler. There are also many philosophical discussions about whether to use “informative” (generally narrow) or “weakly informative” (generally wide) priors. As modelers we should experiment with different priors in simulation to see how and if they change the results significantly. However, we have not found that different “weakly informative” priors changed results much based on posterior distributions of hierarchical DDM parameters. Prior distributions can change posterior distributions if those priors are very narrow, such that values in that parameter’s domain are near impossible (“informative” priors). For instance, a prior of $\beta \sim Normal(.5, .01^{2})$ would restrict the posterior distribution of $\beta$ to be approximately in the domain [.46, .54], within 4 standard deviations on both sides of the mean. We refer readers to a discussion about this topic by Andrew Gelman at https://github.com/stan-dev/stan/wiki/Prior-Choice-Recommendations (Gelman, 2020)

### Assessing posterior distributions

Posterior distributions provide evidence for parameters given the data and specific model architecture. They are influenced by prior distributions, but are often much more influenced by the data itself. In Bayesian analysis, probability is defined as uncertainty. Therefore, we can inspect the posterior distributions themselves to calculate the probability of observing certain values of a parameter given the specific model and data. For instance, the posterior distributions that result from fitting Model 2B to data from Experiment 2 would result in posterior distributions for all parameters, including both effect parameters $\xi _{1j1}$ for experimental condition (1) and $\xi _{1j2}$ for experimental condition (2). We could calculate the probability that each effect parameter is greater than 0.5 (for instance) by finding the proportion of posterior samples that are above 0.5, thus approximating the area under the curves of the posterior distributions, and thus approximating the probability (e.g., evidence) that each effect parameter is greater than 0.5. We can also calculate posterior distributions to answer other questions using a transformation of our parameters. For instance, we can calculate the posterior distribution of the difference between these two effects by matching MCMC samples of the original model fit to get one difference posterior of the quantity $\xi _{1j1} - \xi _{1j2}$ for participant *j*. We can then calculate the probability that $\xi _{1j1} > \xi _{1j2}$ by finding the proportion of posterior samples of the new quantity $\xi _{1j1} - \xi _{1j2}$ that are above zero.

*Bayes factors* (BFs) usually provide the degree of evidence (defined as a probability ratio in Bayesian statistics) for the data given a model. Here we will focus on calculating BFs for a specific case where the parameter value $\lambda$ is exactly equal to some value *x* ($\lambda = x$), compared to the same model where the parameter $\lambda$ can take any other realistic value ($\lambda \ne x$) (Jeffreys, 1961; Kass & Raftery, 1995; Rouder & Morey, 2012; van Ravenzwaaij & Etz, 2021). BFs can also be inverted to give evidence for the more general case ($\lambda \ne x$) compared to the more specific case ($\lambda = x$). Generally, Bayes factors over 3 are considered positive evidence for the numerator model over the denominator model (e.g., the effect is three times more likely under the specific case than the general model) while over 20 is strong evidence (Kass & Raftery, 1995). Bayes factors for parameters of joint models estimated with Bayesian methods can often be estimated using the Savage–Dickey density ratio of the posterior density of parameter $\lambda$ at test value *x* over the prior density of parameter $\lambda$ at test value *x* (Dickey & Lientz, 1970; Verdinelli & Wasserman, 1995; Wagenmakers, Lodewyckx, Kuriyal, & Grasman, 2010; van Ravenzwaaij & Etz, 2021). One example of Savage–Dickey density ratio calculation for hypothetical Experiment 2 is given at the bottom of https://github.com/mdnunez/pyhddmjags/blob/master/model2b_experiment2.py. We have also previously estimated specific Bayes factors (BF1s) of linear relationships between non-decision time $\tau$ parameters and N200 ERP latencies that describe the amount of relative evidence of the effect parameter $\lambda$ being equal to 1 ($\lambda = 1$) to a more general comparison model ($\lambda \ne 1$) using the Savage–Dickey density ratio (Nunez et al., 2019a). These BF1s compared the hypothesis of a “spike” distribution at 1 with no uncertainty in possible effect values ($\lambda = 1$) versus a model with less specific effect values ($\lambda \ne 1$). Note that Bayes factors can describe comparisons of models generally (Etz & Vandekerckhove, 2018; van Doorn et al., 2021). However, for joint modeling purposes we are often interested in comparing point hypotheses to general cases. Other BFs to compare complex models are currently difficult to calculate, and therefore we recommend model comparisons using the methods previously mentioned (e.g., $R^{2}_{\text {pred}}$) for cases other than comparing point hypotheses to general models. Integration of existing work into new packages and further development of these methods will likely make calculating BFs for comparing different models easier in the future (e.g., Gronau et al., 2017; Gronau, Heathcote, & Matzke, 2020)

## Discussion

### Modeling of M/EEG generators

Note that in this paper we assumed summary measures of M/EEG. We could instead model and simulate M/EEG time-series, waveforms, or frequency bands for multiple electrodes/sensors. Considerable work has been conducted to understand the neural generators of M/EEG (e.g., David & Friston, 2003; Nunez & Srinivasan, 2006; Srinivasan, Thorpe, & Nunez, 2013), especially in the field of computational neuroscience. To this end, many researchers in the field of computational neuroscience have built models of M/EEG, scaled up from single-unit neuron activity, the activity of populations of neurons, and the connectivity between neural populations (Daunizeau, David, & Stephan, 2011; Glomb et al., 2021). Developing and simulating these models have been helpful in generating theory to understand how the brain generates M/EEG, and there has been some success relating model parameters to human behavior.

However finding parameter estimates of these models often suffers from the *inverse problem*, known in statistical modeling as *unidentifiability* (Walter, 1987). The inverse problem is relevant across all scientific fields that involve modeling. Inverse problems/unidentifiabilities arise when there is more than one unique parameter set that can describe the data when the generator model is known (Bamber & van Santen, 2000). Therefore, we cannot *invert*, or *find unique parameter estimates*, for a model even though the model can be simulated and we have a good idea of the theoretical concepts. In the context of M/EEG computational modeling, the inverse problem stems from the fact that M/EEG *sources* are not typically confined to a two-dimensional representation of the brain. Instead, they are represented in a three-dimensional manner, while the scalp electrodes (EEG), MEG sensors, or intracranial electrodes (iEEG) exist on curved two-dimensional surfaces. Specifically, these two-dimensional surfaces usually correspond to the scalp, MEG helmet, or electrode strips respectively. Determining these *sources* at specific locations in the brain requires making additional assumptions about the M/EEG generators (e.g., prior information in Bayesian models, see Cai, Sekihara, & Nagarajan, 2018). Unfortunately, these assumptions have proven difficult to validate experimentally due to the invasiveness of surgical procedures, the nature of electrical volume conduction in the head, and the complexity of the brain. Consequently, many studies that explore solutions to the inverse problem for M/EEG rely on comparisons to across algorithms. However, this may change with the advent of new MEG technology (Ilmoniemi & Sarvas, 2019). As a result, some researchers opt to rely on finding only *representative* sources in M/EEG (Nunez et al., 2019b), including in our own work (e.g., Nunez et al., 2019a).

M/EEG records are an extremely rich data source. We usually assume many M/EEG sources in the brain, e.g., dipole sheet, that generate time series data of multiple electrodes, usually 16–256 electrodes in scalp EEG, approximately 300 sensors in MEG, and often > 50 electrodes in intracranial EEG, which are typically sampled at least at 250 Hz. This results in rich multivariate time series data with some spatial information. There are specific M/EEG waveforms, power bands, network interactions, etc. that exist within the data. However, there is no reason to suspect that all useful measures within M/EEG have been studied, and we should expect that there are measures of M/EEG that can predict behavior and cognition that have yet to be found. Even with the advent of machine learning techniques which can explore rich data sets, we expect neurocognitive modeling to reveal more about M/EEG data in the future. Within M/EEG multivariate time series, there are also artifactual components, discussed previously, embedded in both scalp EEG, MEG, and intracranial EEG, but especially scalp EEG and older MEG systems. Thus, including all relevant information for modeling full time-series of M/EEG in a theoretical model can be difficult.

However we do not need to find source estimates of M/EEG to better understand the neurocognitive theory. We could, for instance, translate specific EEG potentials into cognition, EEG potentials like the CPP slope, which in Model 3 is hypothesized to be generated from mean evidence accumulation rate during a trial. We could also instead build models that describe the M/EEG phenomena, such as specific waveforms, while not assuming any particular type of cognition or brain activity. That is, we could describe the observed phenomena and not develop a neurocognitive understanding of M/EEG. This may aid us by allowing measurement noise in observed M/EEG potentials in our models, for instance. One promising method is to simulate EEG time courses by simulating from Morlet wavelet transforms (Bridwell et al., 2018). Specific noise in M/EEG could also be simulated to understand the robustness of the modeling procedure. If specific signals from M/EEG are extracted for joint modeling, such as ERPs or power from a certain frequency band, capturing a full possible range of these signals and contaminants in simulation is beneficial to test the robustness of the procedure (Hawkins et al., 2017).

### The future of joint modeling

We do not consider much of our past work (e.g., Nunez et al., 2015; Nunez et al., 2017; Nunez et al., 2019a) of modeling EEG and human behavior to be “true” joint modeling, in that the our models did not also describe EEG measures. We used hierarchical Bayesian methods, but only assumed simple linear influences of EEG measures on cognitive parameters, similar to Model 2 presented above. These methods could be considered simple *Directed* approaches described by Palestro et al. (2018). Ultimately, as cognitive neuroscientists and cognitive modelers, we would like to develop computational theory that predicts both observed human behavior and EEG dynamics, such as in Model 3. Among the several approaches described by Turner et al. (2017); Palestro et al. (2018), researchers should ultimately seek to use an *Integrative* approach with *simultaneous modeling* of EEG and behavior to test neurocognitive theory. In this way of thinking about model-based cognitive neuroscience, what we have typically performed is *simultaneous joint modeling* with linear connectors between EEG measures and decision-making behavior (a simple *directed* approach in the classes of joint models by Palestro et al., 2018), but not using an *integrative* approach. Furthermore researchers, including ourselves, conducting joint modeling studies have typically fit simultaneous joint models with linear connectors between EEG measures and cognitive parameters (e.g., Frank et al., 2015; Nunez et al., 2017; van Ravenzwaaij et al., 2017; Schubert et al., 2019). Future research should improve upon previous joint modeling work. In the future we wish to use the richness of EEG data and more informative human behavioral measures (e.g., eye-tracking) within joint modeling frameworks to answer important neurocognitive questions. This work will also lead to better *integrative* joint models with possible non-linear connections.

Recently developed model-fitting procedures are making fitting joint models easier. We are particularly excited about algorithms that allow sampling from posteriors of joint models when a likelihood is not available in closed-form or difficult to derive and estimate. One particular promising program is BayesFlow, which finds posterior samples from simulation-based models using invertible neural networks (Radev et al., 2020; Schmitt, Bürkner, Köthe, & Radev, 2022). The first author with colleagues has already used this program with success for *Integrative* joint modeling of single-trial EEG and behavior during decision-making (Ghaderi-Kangavari et al., 2023b). A similar promising and accessible method is to use neural networks to learn approximate likelihoods that can then be used to find posterior distributions of joint models (Fengler, Govindarajan, Chen, & Frank, 2021; Fengler, Bera, Pedersen, & Frank, 2022). In general, we expect future model-fitting procedures to be more flexible in the types of models that can be fit to data, making joint modeling of M/EEG and behavioral data easier to implement.

We expect that joint modeling will gain popularity in various cognitive domains beyond evidence accumulation. This includes the extension of cognitive models of working memory (e.g., Oberauer & Lin, 2017; Oberauer & Lewandowsky, 2019b) and visual metacognition (e.g., Rahnev, 2021). Domain-specific knowledge will be necessary to extend existing cognitive models into neurocognitive models. We aim for this tutorial to serve as a helpful foundation in these advancements.

### Conclusion

We hope this tutorial serves as a guide for those researchers and students interested in joint modeling of M/EEG and behavior. We have covered the possible motivations to perform joint modeling, the definition of joint models, building of joint models, simulating joint models, experimental design, artifactual processes in M/EEG data, specific M/EEG signals, model-fitting implementations, parameter recovery, model comparison, and the future of joint modeling. We have focused our examples on the relationship of scalp-recorded EEG and decision-making. In particular we have used a guiding example of testing the hypothesized relationship of the centro-parietal positivity (CPP) slope to evidence accumulation rate. However, these techniques and principles can easily be applied to other neurocognitive domains and questions using both animal and human electrophysiology. We expect joint modeling to be able to answer questions that cannot be answered with other methods because joint modeling allows direct testing of neurocognitive theory. And we look forward to reading about future research using joint modeling of M/EEG and behavior.

## Exercises

1. Test your EEG artifact identification skills by classifying EEG artifact using independent component analysis. As of August 2023, the website from the makers of ICLabel (Pion-Tonachini et al., 2019) allowed you to practice labeling independent components as “Brain”, “Muscle”, “Eye”, “Heart”, “Line Noise”, “Chan Noise”, or “Other”. Practice at the link https://labeling.ucsd.edu/tutorial/practice and give feedback to improve the ICLabel algorithm at https://labeling.ucsd.edu/labelfeedback.
2. Run the Python or R code from Model 3, rewrite the code in another language (e.g., Julia, see https://github.com/JagsJulia/Jags.jl), and/or rewrite the model code in another Probabilistic Programming Language other than JAGS (e.g., Stan) and then run the code. Plot histograms or density approximations of the response time, accuracy, and CPP slope data for some participants.
3. What model could be compared to Model 3 in order to test the hypothesis that the CPP slope on each trial is a reflection of evidence accumulation? Specifically, what model along with Model 3 could be fit to CPP slopes, response times, and accuracies to test this hypothesis?
4. How could we change Model 3 to test the hypothesis that the CPP slope reflects a *scaled* version of evidence accumulation rate, that is the mean rate of evidence accumulation $\delta$ is not in micro-volts $\mu V$? Assume that the CPP slope could be scaled differently in each participant due to scalp volume conduction differences across participants.
5. Using the given Python simulation of Model 3 as a guide, simulate from Model 1 while assuming that the CPP slopes come from a distribution of $Normal(3,{1}^{2})$ $\mu V$ (micro-volts) per second across trials, that $\theta _{0}$ is equal to .25 seconds, $\theta _{1}$ is equal to .1, $\sigma$ is equal to .1, $\gamma _{0}$ is equal to 0, and $\gamma _{1}$ is equal to .3. Does this model produce similar (and in this case of Model 3, more realistic) values of response times *r* and accuracies *x* across trials *n* compared to Model 3?

Solutions are located after the *Appendix* of this document.

## Further readings

Here we present a reading list containing articles and books that offer discussions on a range of relevant subjects within neurocognitive modeling. The reading list follows the same section headers as outlined in the main text.

*1. Motivation to model*

- Blohm, G., Kording, K. P., & Schrater, P. R. (2020). A how-to-model guide for neuroscience. *eNeuro,* *7*(1).
- Forstmann, B. U., & Wagenmakers, E.-J. (Eds.). (2015). *An introduction to model-based cognitive neuroscience*. New York, NY: Springer, New York.
- Wang, Z. J., & Busemeyer, J. R. (2021). *Cognitive choice modeling*. Cognitive choice modeling. Cambridge, MA, US: The MIT Press.

*2. Models to describe joint data*

- Harris, A., & Hutcherson, C. A. (2022). Temporal dynamics of decision-making: A synthesis of computational and neurophysiological approaches. *WIREs Cognitive Science,* *13*(3), e1586.
- Palestro, J. J., Bahg, G., Sederberg, P. B., Lu, Z.-L., Steyvers, M., & Turner, B. M. (2018). A tutorial on joint models of neural and behavioral measures of cognition. *Journal of Mathematical Psychology,* *84*, 20–48.
- Turner, B. M., Forstmann, B. U., & Steyvers, M. (2019). *Joint models of neural and behavioral data*. Computational approaches to cognition and perception: Springer International Publishing.

*3. Experimental manipulations and experimental design*

- Jensen, K. M., & MacDonald, J. A. (2023). Towards thoughtful planning of ERP studies: How participants, trials, and effect magnitude interact to influence statistical power across seven ERP components. *Psychophysiology,* *60*(7), e14245.

*4. Collection and preprocessing of M/EEG for joint modeling*

- Boudewyn, M. A., Erickson, M. A., Winsler, K., Ragland, J. D., Yonelinas, A., Frank, M., ... Carter, C. S. (2023). Managing EEG studies: How to prepare and what to do once data collection has begun. *Psychophysiology* (pp. e14365).
- Cohen, M. X. (2014). *Analyzing neural time series data: Theory and practice*. MIT Press.
- Luck, S. J. (2014). *An introduction to the event-related potential technique, Second edition*. MIT Press.
- Luck, S. J. (2022). *Applied event-related potential data analysis*. LibreTexts.
- Nunez, M. D., Nunez, P. L., & Srinivasan, R. (2016). Electroencephalography (EEG): Neurophysics, experimental methods, and signal processing. In H. Ombao, M. Linquist, W. Thompson, & J. Aston (Eds.), *Handbook of neuroimaging data analysis* (pp. 175–197). Chapman & Hall/CRC.

*5. Implementing model-fitting procedures and estimating parameters*

- Baribault, B. & Collins, A. G. E. (2023). Troubleshooting Bayesian cognitive models. *Psychological Methods*.
- Etz, A., & Vandekerckhove, J. (2018). Introduction to Bayesian inference for psychology. *Psychonomic Bulletin & Review,* *25*(1), 5–34.
- Farrell, S., & Lewandowsky, S. (2018). *Computational modeling of cognition and behavior*. Cambridge: Cambridge University Press.
- Lee, M. D. & Wagenmakers, E.-J. (2014). *Bayesian cognitive modeling: A practical course*. Cambridge University Press.
- Myung, J. I., & Pitt, M. A. (2018). Model comparison in psychology. *Stevens’ handbook of experimental psychology and cognitive neuroscience,* *5*, 85–118.
- McElreath, R. (2018). *Statistical rethinking: A Bayesian course with examples in R and Stan*. CRC Press.
- Palestro, J. J., Bahg, G., Sederberg, P. B., Lu, Z.-L., Steyvers, M., & Turner, B. M. (2018). A tutorial on joint models of neural and behavioral measures of cognition. *Journal of Mathematical Psychology,* *84*, 20–48.
- Schad, D. J., Betancourt, M., & Vasishth, S. (2021). Toward a principled Bayesian workflow in cognitive science. *Psychological Methods,* *26*(1), 103–126.
- Wilson, R. C., & Collins, A. G. (2019). Ten simple rules for the computational modeling of behavioral data. *eLife,* *8*, e49547.

*6. Discussion*

- Bridwell, D. A., Cavanagh, J. F., Collins, A. G. E., Nunez, M. D., Srinivasan, R., Stober, S., & Calhoun, V. D. (2018). Moving beyond ERP components: A selective review of approaches to integrate EEG and behavior. *Frontiers in Human Neuroscience,* *12*, 106.

## Appendix

### An illustrative implementation of neurocognitive modeling

The following code is a slightly modified and annotated version of Python code at this location: https://github.com/mdnunez/pyhddmjags/blob/master/simpleCPP_test.py. For this tutorial, we will walk through the process of simulating data from Model 3, writing the corresponding JAGS code for the model, and fitting the model to our simulated data.

#### Simulating data

In Code Block 2, we first start by generating choice, response times, and CPP slopes from Model 3 (for a brief description, see below, or for more in-depth information, refer to the main text). Lines 3–11, first import the necessary modules, as well as a custom module named pyhddmjagsutils. In lines 19–25, we specify the number of participants (nparts = 100) and trials (ntrials = 100) we would like to simulate. The total number of trials is then specified as N = ntrials * nparts. Lines 30–40 randomly initialize model parameters from uniform distributions with specified bounds. Lines 47–57 then loop through each participant, simulating their choice and response times using the simulratcliff function from pyhddmjagsutils, and CPP slopes from a normal distribution. simulratcliff is exactly the code we showed previously for simulating choice and response time data in Code Block 1 in the main text.

The remainder of Code Block 2 initializes and stores all the generated data and parameters in a dictionary called ‘genparam‘.

#### Specifying a joint model

In this section, we describe how to specify a joint model. Specifically, we discuss how JAGS and Python handler code can be used to write models. In our example, the JAGS code is written as a string within Python and saved into a file to be called during sampling. However, you can also specify your JAGS file separately. In the worked example we focus on Model 3 as discussed the main text. We briefly restate it convenience here. Recall that Model 3 tries to describe the cognitive parameters through the EEG measures themselves, allowing us to test the underlying computational role of the CPP slope in cognition.

12 $$\begin{aligned} (r_{ij},x_{ij})&\sim DDM(\delta _{j} ,\tau _{j}, \alpha _{j}, \beta _{j}, \eta _{j}) \end{aligned}$$

13 $$\begin{aligned} c_{ij}&\sim Normal(\delta _{j} , \ \sigma _{j}^{2}) \end{aligned}$$

Let CPP slope be denoted by the variable *c*, accuracy be denoted by *x*, and response times be denoted by *r*. Note that *x*, *r*, and *c* can vary on every trial *i* and by participant *j*. In terms of parameters we are interested in the five cognitive parameters of a DDM, $\delta$, $\tau$, $\alpha$, $\beta$, $\eta$ and one computational parameter $\sigma$ that vary by participant *j*. In Model 3, the mean of each trial’s CPP slope *c* is described by the drift rate for each participant *j*. Lastly, we assume, for model *fitting*, that there is no extra trial-to-trial variability in drift rate (e.g., assuming $\eta$ = 0), due to recovery work by Dutilh et al. (2019) and our own recovery work above showing that assuming this parameter is not necessarily to estimate the other parameters.

The JAGS code consists of two sections: the first defines our priors on the model parameters, and the second defines the likelihood function. Lines 9–24 loop through each participant, specifying priors on each of the model parameters. Because we are fitting our model within a Bayesian framework, we need to specify our priors. For simplicity, we will use the normal distribution $\mathcal {N}$, parameterized by mean and variance respectively, and the truncated normal distribution with boundaries *a* and *b* denoted by $\in (a,b)$.

14 $$\begin{aligned} \alpha _{j} \sim \mathcal {N}(1,.5^{2}) \in (0,3) \end{aligned}$$

15 $$\begin{aligned} \tau _{j} \sim \mathcal {N}(.5,.25^{2}) \in (0,1) \end{aligned}$$

16 $$\begin{aligned} \beta _{j} \sim \mathcal {N}(.5,.25^{2}) \in (0,1) \end{aligned}$$

17 $$\begin{aligned} \delta _{j} \sim \mathcal {N}(0,2^{2}) \end{aligned}$$

18 $$\begin{aligned} \sigma _{j} \sim \mathcal {N}(1,.5^{2}) \in (0,3) \end{aligned}$$

For a discussion on prior selection in cognitive modeling, see Lee and Vanpaemel (2018), and for a discussion specific to DDMs, Matzke and Wagenmakers (2009); Tran, van Maanen, Heathcote, and Matzke (2021). One thing to note is that JAGS parameterizes the normal distributions in terms of precision rather than standard deviation. Thus, throughout the model specification, we use the pow function to convert our standard deviations into precision parameters.

Next, Lines 29–36 define our likelihood function. Following Eq. (1) and (2), the likelihood function loops through each trial to generate samples from the joint posterior distribution of the model parameters, conditioned on the simulated data. This can be written more formally as:

19 $$\begin{aligned}&p(\delta ,\tau , \alpha , \beta , \sigma | y,c) \propto \mathcal {L} \nonumber \\&(\delta ,\tau , \alpha , \beta |y)\mathcal {L}(\delta , \sigma |c)p(\delta ,\tau , \alpha , \beta , \sigma ) \end{aligned}$$

This predicted joint distribution of the data, conditional on all parameters, is then the first passage time distribution of a Wiener process with constant drift ($\eta = 0$) and a normal distribution to describe the mean CPP slopes. Note that for each trial $i$, subject $j$, the observed accuracy $x_{ij}$ and response time $r_{ij}$ are combined into a two-element vector $y_{ij}$.

Note that this model could be easily extended to a *hierarchical* model with changed *priors* and *hyperpriors*. We hierarchical model examples in the Python https://github.com/mdnunez/pyhddmjags and R https://github.com/kiante-fernandez/Rhddmjags repositories, see also *Further readings* of *Implementing model-fitting procedures and estimating parameters*, especially by Lee and Wagenmakers (2014) for a discussion on hierarchical models with examples in JAGS.

#### Fitting a joint model to data

Once we have specified our joint model, we can fit it to the data. Specifically, we discuss how the Python handler code can be used to fit models to our simulated data. At this point, we will assume that you have installed the JAGS software and the necessary extension for sampling from the Wiener distribution in JAGS (for details, see Wabersich & Vandekerckhove, 2014). The goal of the following code is to sample from the joint posterior distribution specified in the Code Block and in Eq. 19 above.

We start by setting the seed in Line 3. Setting the seed in statistical simulations ensures that the sequence of random numbers generated is reproducible across different runs and machines. Lines 6–7 load the two JAGS extensions, one for the Wiener distribution and the other for conveniently calculating the deviance information criterion (DIC), which is a method for comparing the relative fit of a set of models (for a discussion on model comparisons, see Myung & Pitt, 2018). Lines 10–12 describe how many chains to use during sampling, how many to discard for burn-in, and the total number of samples to draw from our posterior, respectively. Line 15 defines which parameters we are interested in collecting samples from one the sampling is finished. Lines 17–25 use our simulated data to define the variables we would like pass to the JAGS code.

Lines 27–43 set the initial values for JAGS MCMC sampler (for a discussion on MCMC, see van Ravenzwaaij, Cassey, & Brown, 2018). While JAGS will define values based on the priors by default, setting initial values can help with convergence and efficiency in sampling our joint posterior. On the other hand, poorly selected starting values may result in slow or failed convergence, leading to unreliable estimates. For example, in our case, we know that ndt for a given participant should no more than their fastest response time, and thus we can randomly initialize the ndt parameter from uniform distributions with participant-specified bounds that reflect that. Lastly Lines 44-49 take in as input all of the variables specified above to draws samples the joint posterior distribution.

We have omitted a few technical details associated with employing Bayesian methods to fit computational models. For more comprehensive treatments, we suggest referring to our *Implementing model-fitting procedures and estimating parameters* section in the *Further readings*.

#### Conclusions

In this appendix, we have showcased only one example implementation of neurocognitive modeling. In the tutorial repositories, we have designed several illustrative examples in Python: https://github.com/mdnunez/pyhddmjags and R: https://github.com/kiante-fernandez/Rhddmjags that follow a similar format to what we have explained here.

## Solutions

1. Labeling feedback given by the ICLabel website.
2. Plotting of histograms are left to the reader. Plots of density approximations should approximately reproduce the results in Fig. 2. The code for this figure is given in https://github.com/mdnunez/pyhddmjags/blob/master/simpleCPP_sim.py. For the corresponding translation in R see: https://github.com/kiante-fernandez/Rhddmjags/blob/main/inst/templates/CPP_sim-example.R.
3. A comparison model would be similar to Model 3 other than the fact that CPP slopes *c* per trial *i* are not generated by drift rates $\delta$, a cognitive parameter that describes the mean rate of evidence accumulation.
  20 $$\begin{aligned}&(r_{ij},x_{ij}) \sim DDM(\delta _{j} ,\tau _{j}, \alpha _{j}, \beta _{j}, \eta _{j}) \end{aligned}$$
  21 $$\begin{aligned}&c_{ij} \quad \quad \quad \sim Normal(\phi _{j} , \sigma _{j}^{2}) \end{aligned}$$
  Note that this comparison model has an additional parameter per participant, $\phi$, compared to Model 3. $\phi$ is just the mean CPP slope across trials.
4. We could add an addition scaling parameter $\psi$ in the second equation that can change based on participant *j*:
  22 $$\begin{aligned} c_{ij} \sim Normal(\psi _{j}\delta _{j} , \sigma _{j}^{2}) \end{aligned}$$
5. This model produces normally distributed response times, while Model 3 produces response time distributions with right skews. Apart from very specific conditions (e.g., when facing a strict deadline that puts one under severe time pressure), response time distributions are usually right skewed, making Model 3 more plausible.

## Declarations

### Open practices statement

All the code and data simulations from this work are given in the following repositories as of July 2023: https://github.com/mdnunez/pyhddmjags and https://github.com/kiante-fernandez/Rhddmjags. These analyses were not preregistered.
